# Dynamics of Th1/Th17 responses and antimicrobial pathways in leprosy skin lesions

**DOI:** 10.1172/JCI190736

**Published:** 2025-06-26

**Authors:** Priscila R. Andrade, Feiyang Ma, Jing Lu, Jaime de Anda, Ernest Y. Lee, George W. Agak, Craig J. Dobry, Bruno J. de Andrade Silva, Rosane M.B. Teles, Lilah A. Mansky, Jonathan Perrie, Dennis J. Montoya, Bryan D. Bryson, Johann E. Gudjonsson, Gerard C.L. Wong, Euzenir N. Sarno, Matteo Pellegrini, Robert L. Modlin

**Affiliations:** 1 Division of Dermatology, Department of Medicine, David Geffen School of Medicine, UCLA, Los Angeles, California, USA.; 2 Department of Molecular, Cell, and Developmental Biology and; 3 Department of Bioengineering, UCLA, Los Angeles, California, USA.; 4 Department of Dermatology, UCSF, San Francisco, California, USA.; 5 Department of Dermatology, University of Michigan, Ann Arbor, Michigan, USA.; 6 Broad Institute of MIT and Harvard, Cambridge, Massachusetts, USA.; 7 Department of Biological Engineering, MIT, Cambridge, Massachusetts, USA.; 8 Leprosy Laboratory, Oswaldo Cruz Foundation, Rio de Janeiro, Brazil.; 9 Department of Microbiology, Immunology and Molecular Genetics, UCLA, Los Angeles, California, USA.

**Keywords:** Immunology, Infectious disease, Adaptive immunity, Bacterial infections, Innate immunity

## Abstract

**BACKGROUND:**

Reversal reactions (RRs) in leprosy are acute immune episodes marked by inflammation and bacterial clearance, offering a model to study the dynamics of host responses to *Mycobacterium leprae*. These episodes are often severe and difficult to treat, frequently progressing to permanent disabilities. We aimed to characterize the immune mechanisms and identify antimicrobial effectors during RRs.

**METHODS:**

We performed RNA-Seq on paired skin biopsy specimens collected from 9 patients with leprosy before and at RR diagnosis, followed by differential gene expression and functional analysis. A machine-learning classifier was applied to predict membrane-permeabilizing proteins. Antimicrobial activity was assessed in *M*. *leprae*–infected macrophages and axenic cultures.

**RESULTS:**

In the paired pre-RR and RR biopsy specimens, a 64-gene antimicrobial response signature was upregulated during RR and correlated with reduced *M*. *leprae* burden. Predicted upstream regulators included IL-1β, TNF, IFN-γ, and IL-17, indicating activation of both the Th1 and Th17 pathways. A machine-learning classifier identified 28 genes with predicted membrane-permeabilizing antimicrobial activity, including S100A8. Four proteins (S100A7, S100A8, CCL17, and CCL19) demonstrated antimicrobial activity against *M*. *leprae* in vitro. Scanning electron microscopy revealed membrane damage in bacteria exposed to these proteins.

**CONCLUSION:**

RR is associated with a robust antimicrobial gene program regulated by Th1 and Th17 cytokines. We identified potentially novel host antimicrobial effectors that showed activity against *M*. *leprae*, suggesting potential strategies to bolster Th1 and Th17 responses for combating intracellular mycobacterial infections.

**FUNDING:**

NIH grants R01 AI022553, R01 AR040312, R01 AR073252, R01 AI166313, R01 AI169526, P50 AR080594, and 4R37 AI052453-21 and National Science Foundation (NSF) grant DMR2325840.

## Abstract 2

Reversal reactions in leprosy reveal dynamic immune responses. We identified an antimicrobial gene signature, regulated by Th1/Th17 cytokines, highlighting potential antimicrobial effectors and therapeutic targets for improving mycobacterial infection outcomes.

## Graphical abstract

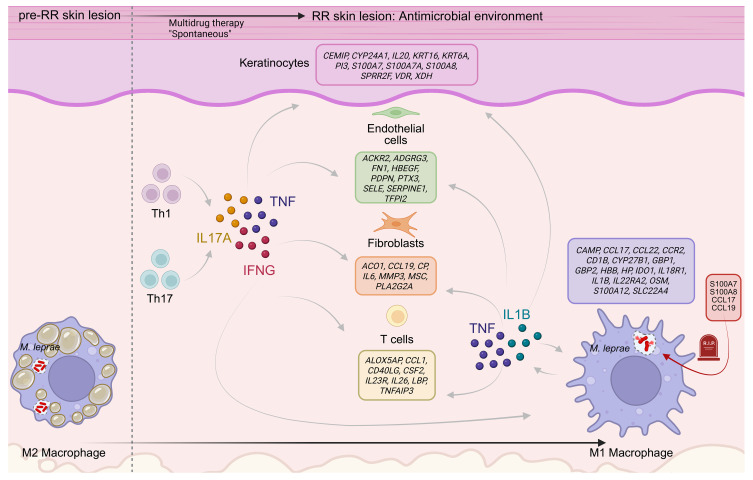

## Introduction

Central to an effective host defense strategy against intracellular pathogens is the interaction of the innate and adaptive immune systems to mount a robust cell-mediated immune (CMI) response involving antimicrobial mechanisms. Leprosy provides a human disease model to investigate such mechanisms, as the clinical manifestations correlate with the immune response to the intracellular bacterium *Mycobacterium leprae* (1). The CMI response is strongest in individuals in the self-limiting tuberculoid pole (T-lep), as evidenced by the Th1 cytokine profile production (2) and the vitamin D–dependent antimicrobial pathway induced by IFN-γ that can program macrophages to kill intracellular bacteria (3, 4). As a result, these patients exhibit few, often self-healing skin lesions, in which *M*. *leprae* bacilli are rarely found. Conversely, individuals with the progressive lepromatous pole (L-lep) are susceptible to disseminated infection, displaying numerous skin lesions loaded with bacilli due to an ineffective CMI response, and instead showing high antibody titers, Th2 cytokine production (2), and phagocytic macrophages permissive to infection (3).

Individuals with leprosy can undergo acute inflammatory episodes known as reactions that ignite intense immune responses followed by severe outcomes. A type I reaction, or reversal reaction (RR), consists of a series of dynamic changes to the patient’s immunological state that occur either spontaneously before, during, or after chemotherapy, typically with a shift from the L-lep toward the T-lep pole of the spectrum (5–7). RR presents clinically with the sudden appearance of new inflammatory skin lesions or the exacerbation of existing ones with the presence of erythema and edema, often associated with peripheral nerve impairment (6, 8). Histologically, RR skin lesions exhibit organized granulomas similar to those found in T-lep lesions, with the presence of intercellular edema and epithelioid cell populations (8, 9). Patients exhibit an enhanced CMI response to *M*. *leprae* antigens associated with the reduction or clearance of bacilli in their skin lesions (10, 11), the influx of Th CD4^+^ and cytotoxic CD8^+^ T cell populations (12), a shift from a Th2 to a Th1 profile (10, 12, 13), plasticity from M2-like to M1-like macrophages (3), as well as an increase in IFN-γ–induced genes and a decrease in IFN-β–triggered responses including IL-10 production (3, 4, 8).

The initial host response against mycobacterial infection includes triggering of the innate immune response involving antimicrobial mechanisms, pattern recognition receptor pathway activation (14, 15), vitamin D pathway induction (3, 16, 17), production of antimicrobial peptides (AMPs) (18–20), and initiation of autophagy (21, 22). This innate response also leads to subsequent activation of the adaptive immune response that leads to CMI. An effective CMI response against mycobacteria is dependent on the T cell release of antimicrobial effector molecules, as well as induction of antimicrobial effector mechanisms in infected macrophages. Th1 cell release of IFN-γ (4, 20, 21) can induce antimicrobial activity against *M*. *leprae* and *M*. *tuberculosis* in human macrophages via the vitamin D-dependent pathway that results in autophagy, phagosomal maturation, and production of the AMP cathelicidin (4, 21, 22). Human CD8^+^ cytotoxic T cells expressing the cytotoxic granule proteins granzyme B, perforin, and granulysin have been linked to host defense in leprosy and tuberculosis (23, 24), with both granulysin and granzyme B having direct antimycobacterial activity (25, 26). In addition, Th17 cells can release IL-26, which has direct antimicrobial activity against *M*. *leprae* and *M*. *tuberculosis* (27–29).

Longitudinal studies of patients before and at the onset of RRs have been conducted previously (9, 13, 30–35), mostly examining the immune response in the peripheral blood, with some assessing a small number of genes or proteins in patient lesions. In this study, we sought to uncover the dynamics of innate and adaptive antimicrobial mechanisms at the site of disease by investigating the dynamic changes in the RR transcriptome in paired skin biopsy samples collected from patients before and at the onset of RR.

## Results

### Differential gene expression analysis shows the dynamic change in antimicrobial gene expression during RR.

To study the dynamic changes in immune response genes at the site of infection associated with the onset of a CMI response in RRs, we performed RNA-Seq on paired skin biopsy specimens obtained from 9 patients with leprosy at the time of diagnosis with multibacillary disease (pre-RR) and at the clinical presentation of the RR (Supplemental Figure 1; supplemental material available online with this article; https://doi.org/10.1172/JCI190736DS1). The inclusion of these patients in our study was supported by clinical examination and histopathologic correlation by experienced leprologists at the Oswaldo Cruz Foundation (Supplemental Figure 2).

**Figure 1:**
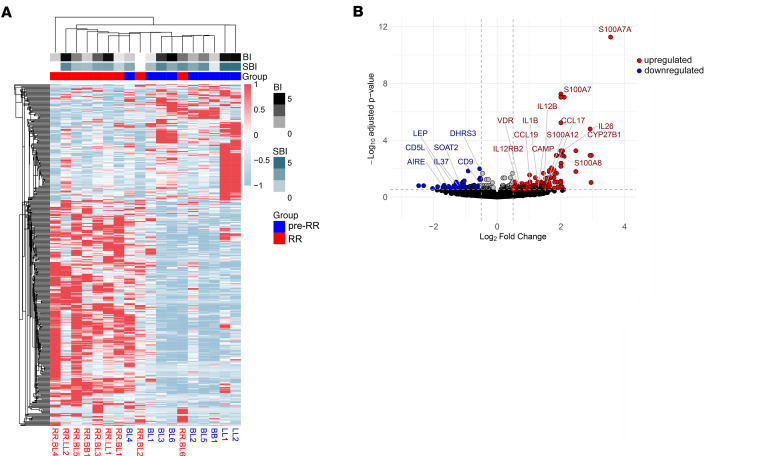
Differential gene expression analysis of RR versus pre-RR groups. (**A**) Heatmap displaying expression *z* scores for the 404 differentially expressed genes (*Padj* < 0.3) in RR versus pre-RR specimens, representing high (red) and low (light blue) expression levels. Samples were clustered using Euclidean distance and median linkage. (**B**) Volcano plot of the differential gene expression analysis showing RR-upregulated (red) and downregulated (blue) genes. The paired inverted β binomial test was used to perform differential gene expression analysis. The relevant genes are annotated in the plot.

We isolated total RNA from 18 skin specimens (*n* = 9 pre-RRs and *n* = 9 RRs) (Supplemental Table 1), depleted human ribosomal RNA to enrich the samples for mRNAs, prepared stranded libraries and submitted the samples for sequencing. Dimensionality reduction on the transcriptome data did not show clear separation of the pre-RR and RR samples into distinct clusters, likely due to the shared characteristics of the paired individuals, as seen previously (34) (Supplemental Figure 3). To uncover differences between the RR and pre-RR transcriptomes, we conducted a paired differential gene expression analysis. We identified 404 genes (adjusted *P* value [*Padj*] < 0.3) that were differentially expressed between the RR versus pre-RR groups, of which 200 genes (log_2_ fold change [FC] > 0.5, *Padj* < 0.3) were upregulated in RRs, whereas 79 genes were downregulated (log_2_ FC < –0.5, *Padj* < 0.3) (Supplemental Data File 1). Hierarchical clustering analysis using the 404 differentially expressed genes showed segregation of the samples into 2 distinct clusters of 9 samples each, 1 predominantly from pre-RR and the other from RR patients. The RR cluster contained 1 pre-RR sample, BL4, while the pre-RR cluster contained 1 RR sample, RR.BL6 (Figure 1A). Patients BL4 and BL6 developed RRs at 2.4 and 9.9 months after leprosy diagnosis, respectively (Supplemental Table 1). Histological review of all the biopsy specimens revealed that pairs BL4→RR.BL4 and BL6→RR.BL6 had the least pronounced differences between pre-RR and RR states among all 9 patients, providing one possible explanation for being outliers in the hierarchical clustering analysis.

A volcano plot of the differentially expressed genes revealed that the RR lesions highly expressed *CAMP*, *CYP27B1*, *VDR*, and *IL1B*, elements of the vitamin D–dependent antimicrobial pathway (16, 19), as well as *IL26*, which encodes an antimicrobial protein released by IL-1β–activated IL-1R1^+^ Th17 cells (28, 36). RR specimens also expressed *S100A12*, which encodes an antimicrobial protein induced by TLR2/1L and IFN-γ in human macrophages (20), as well as *IL12B* and *IL12RB2*, known to be involved in host defense against leprosy (37). On the other hand, pre-RR lesions expressed genes that contribute to immunosuppression (*IL37*, *AIRE*) (38, 39) and genes involved in lipid metabolism or foamy macrophage biology (*DHRS3*, *SOAT2*, *CD5L*, *CD9*, *LEP*) (40–44) (Figure 1B).

**Figure 2:**
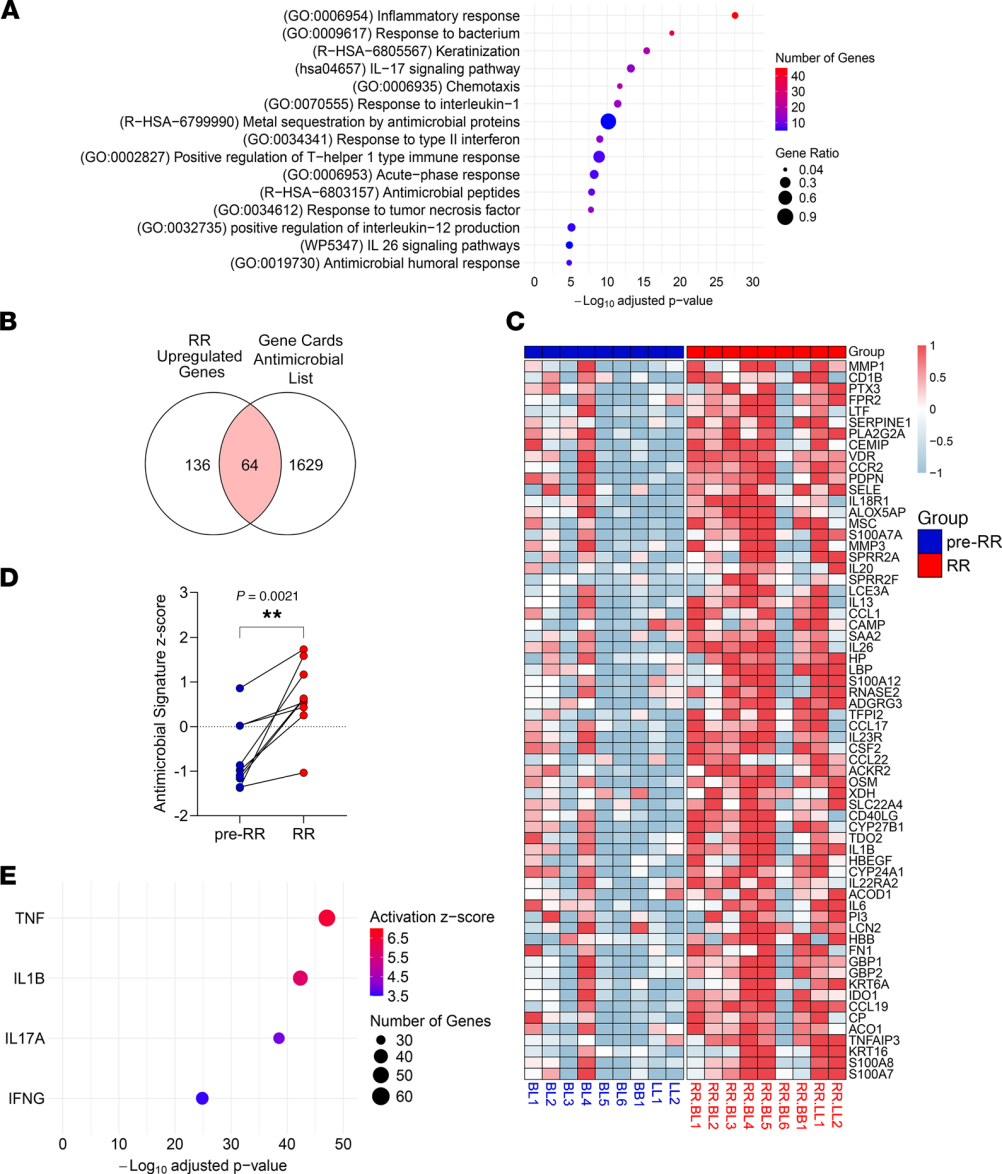
Functional analysis of RR-upregulated genes. (**A**) Dot plot of selected host defense functional pathways enriched (–log_10_ *Padj* >1.3 = *Padj* < 0.05) in the RR-upregulated gene signature. (**B**) Venn diagram depicting overlap between the GeneCards database antimicrobial gene signature (*n* =1,693) and the RR-upregulated genes (*n* = 200). (**C**) Heatmap showing the expression of 64 antimicrobial genes in each patient before (pre-RR) and at RR clinical onset (RR). (**D**) Antimicrobial response signature *z* score for each patient before and at RR clinical onset. Statistical analyses were performed in GraphPad Prism 9.12 using a paired 2-tailed *t* test. **P < 0.01. (**E**) Dot plot showing the IPA UPR analysis of the 64 antimicrobial genes upregulated in RR skin lesions.

Functional analysis of the RR upregulated gene signature using Metascape (45) showed significant enrichment for host defense pathways such as the “inflammatory response” (–log_10_ *Padj* = 27.6), “response to bacterium” (–log_10_ *Padj* = 18.8), “IL-17 signaling pathway” (–log_10_ *Padj* = 13.3, and “chemotaxis” (–log_10_ *Padj* = 11.7), reflecting the emergence of host defense mechanisms at the site of disease (Figure 2A). In addition, the RR pathways also included “metal sequestration by antimicrobial proteins” (–log_10_ *Padj* = 10.1) and “antimicrobial peptides” (–log_10_ *Padj* = 7.8).

To elucidate the antimicrobial response in RRs, we overlapped the RR-upregulated, 200-gene signature with a list of 1,693 genes encoding proteins involved in antimicrobial responses from the Gene Cards database, which identified a 64-gene antimicrobial response signature (enrichment –log_10_ *P* = 15.9) (Supplemental Data File 2, Figure 2B, and Supplemental Figure 4). A heatmap showing the expression of all 64 genes in the paired patient samples showed the dynamic upregulation of antimicrobial genes from pre-RR to RR (Figure 2C), despite the variable expression levels at the time of the pre-RR state. We calculated an antimicrobial response signature score by averaging the expression of all the 64 genes in each patient and then deriving *z* scores. Our analysis showed a significant increase of the antimicrobial response signature *z* score in the RR group (mean = 0.65, SEM ± 0.25) when compared with the pre-RR specimens (mean = –0.65, SEM ± 0.27) (Figure 2D). Correlation analyses between each patient’s antimicrobial response signature *z* scores and clinical variables listed in Supplemental Table 1 — including sex, age, multidrug therapy (MDT) duration, number of RR lesions, and time from leprosy diagnosis to RR onset — revealed no significant association between antimicrobial gene expression and these clinical features (data not shown). To validate the association of the 64-gene antimicrobial response signature in RRs versus pre-RRs with the self-limiting versus progressive forms, we mined other leprosy skin lesion RNA-Seq datasets and signatures (Supplemental Data File 3). Overall, 48 genes of the 64-gene RR antimicrobial response signature were confirmed in the self-limiting forms (T-lep and RR) of other leprosy datasets.

Upstream regulator (UPR) analysis of the 64-gene antimicrobial response signature using Ingenuity Pathway Analysis (IPA) software revealed that innate and adaptive immune cytokines were among the most significant upstream regulators, each targeting a high number of RR antimicrobial genes within the signature. Notable UPRs of this signature included *TNF* (–log_10_ *Padj* = 47.1), *IL1B* (–log_10_ *Padj* = 42.3), *IL17A* (–log_10_ *Padj* = 38.5), and *IFNG* (–log_10_ *Padj* = 24.8) (Figure 2E). The UPR analysis showed that 57 of the 64-gene antimicrobial response signature were regulated by these cytokines, with 44 RR antimicrobial genes (77.2%) being induced by either innate (*TNF* or *IL1B*) or adaptive cytokines (*IFNG* or *IL17A*). IL-1β and TNF were shown to exclusively induce the expression of 12 genes, while IFN-γ was the single inducer of only 1 antimicrobial gene in the signature (Supplemental Figure 5A). We mined an independent leprosy single-cell RNA-Seq (scRNA-Seq) dataset composed of RR and L-lep skin lesions (GSE151528) (18) and found that IL1B mRNA expression in RR lesions was restricted to myeloid cells, while TNF was expressed by both myeloid and T cells, with higher expression in myeloid cells. (Supplemental Figure 5B). *IFNG* expression was primarily detected in T cells with both *IFNG* and *TNF* predominantly expressed in the Th17 cells and RR cytolytic T lymphocytes (RR CTL) subpopulations (Supplemental Figure 5, C and D). *IL17A* mRNA was weakly expressed, however Th17 cells have been detected in RR lesions by scRNA-Seq (18, 46) according to the key markers *RBPJ*, *RORA*, *RORC*, *IL23R*, and *CCL20* (18), and IL-17 protein has been detected in T-lep skin lesions (47, 48).

**Figure 3:**
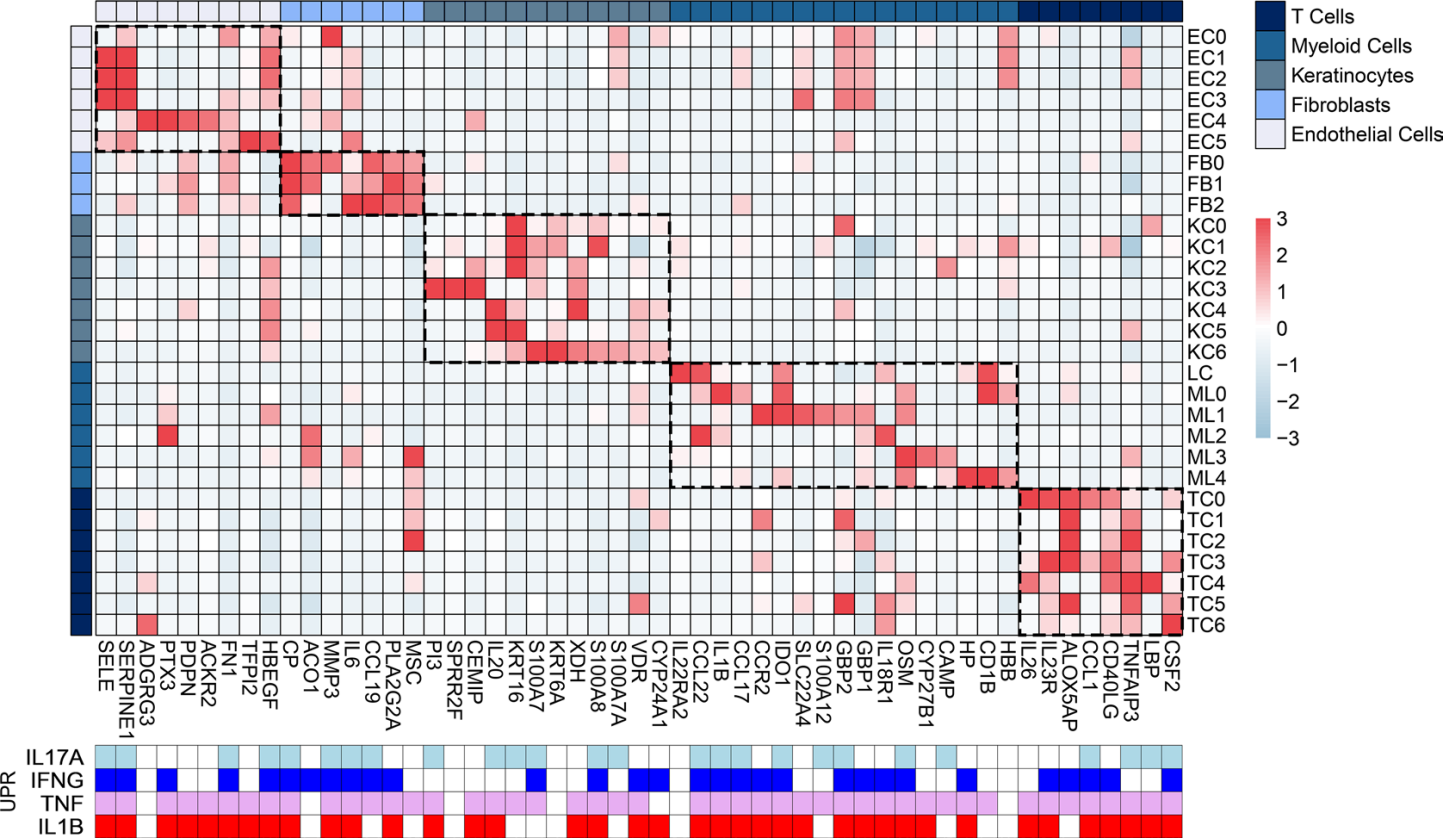
Different cell populations in RR skin lesions express the RR antimicrobial response signature. Heatmap of average expression *z* scores for 53 of the 64 genes from the RR antimicrobial response signature (*z* score >2) detected in RR cell types defined by scRNA-Seq (GSE151528). The heatmap’s red-to-blue color scale indicates high to low expression. Cell type subclusters represent T cells (TC), myeloid cells (LC and ML), keratinocytes (KC), fibroblasts (FB), and endothelial cells (EC). The regulation of the antimicrobial genes (*z* score >2) by their respective UPRs is depicted as a heatmap at the bottom in light blue (*IL17A*), dark blue (*IFNG*), violet (*TNF*), and red (*IL1B*).

We also identified cells expressing the 64 antimicrobial response genes in the RR skin lesions by calculating the average expression *z* score for these genes in the scRNA-Seq cell clusters detected in the RR and L-lep skin lesions (18). Of the 64 antimicrobial genes, 53 were found to have a *z* score above 2 in at least 1 cell subtype in the RR samples spanning myeloid cells, keratinocytes, endothelial cells, T cells, and fibroblasts (Figure 3). *TNF*, one of the top UPRs of the RR antimicrobial response, regulated 54 of the 64 antimicrobial genes. Of these, 46 were detected in the leprosy scRNA-Seq dataset (18), with elevated expression (*z* score >2) observed in endothelial cells (*n* = 8), fibroblasts (*n* = 6), keratinocytes (*n* = 9), myeloid cells (*n* = 15), and T cells (*n* = 8), indicating a broad effect of *TNF* across multiple skin cell populations during RRs. Similarly, among the 32 RR antimicrobial genes regulated by IL-17, we identified 26 in endothelial cells (*n* = 4), fibroblasts (*n* = 4), keratinocytes (*n* = 6), myeloid cells (*n* = 8), and T cells (*n* = 4). This widespread regulatory effect was also observed for IFN-γ and IL-1β, further supporting their role in shaping the RR skin lesion environment (Figure 3). Together our results indicate the contribution of both the innate and adaptive branches of the host immune response to the dynamic increase in the antimicrobial gene signature by different skin cell populations during the host response in RRs, including the involvement of a robust Th17 helper response.

### The RR antimicrobial response gene signature is detected in patients with T-lep and inversely correlates with M. leprae burden.

**Figure 4:**
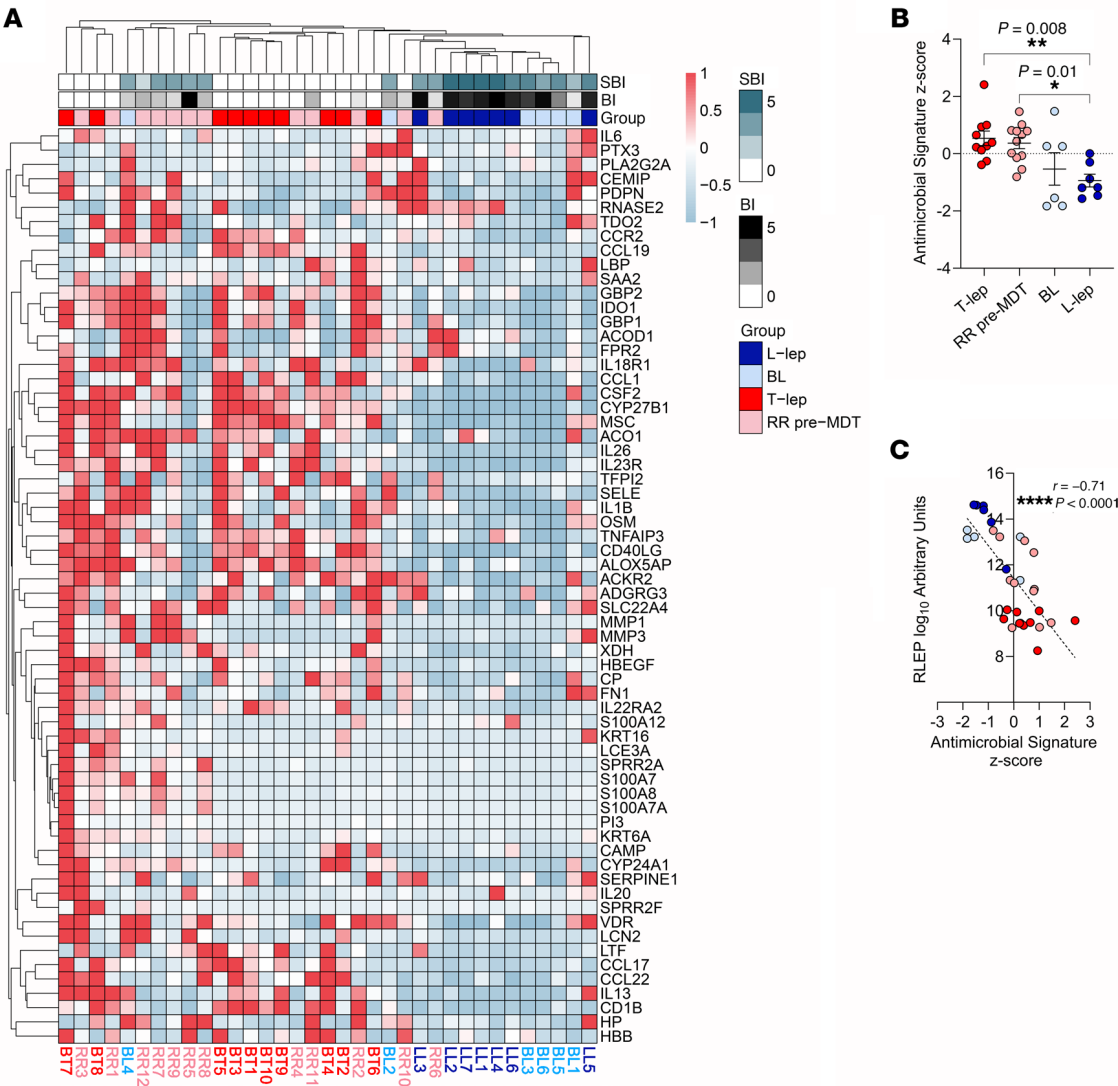
The RR antimicrobial response signature is more highly expressed in T-lep and RR pre-MDT patients and negatively correlates with bacillary load. (**A**) Heatmap displaying expression *z* scores for the 64 RR antimicrobial genes in leprosy clinical forms, with red to light blue color scale indicating high to low expression. T-lep, RR pre-MDT, BL, and L-lep samples were grouped by hierarchical clustering using Canberra distance and the McQuitty linkage method. (**B**) Plot showing the antimicrobial response signature *z* scores for each patient from the T-lep (*n* = 10), RR pre-MDT (*n* = 12), BL (*n* = 6), and L-lep (*n* = 7) groups. Data represent the mean ± SEM. (**C**) Correlation analysis between *RLEP* expression and antimicrobial response gene signature *z* scores for each patient from the T-lep (red), RR pre-MDT (pink), BL (light blue), and L-lep (blue) groups. Statistical analyses were performed in GraphPad Prism 9.12 using ordinary 1-way ANOVA followed by Tukey’s multiple-comparison test (**B**) and Spearman’s correlation coefficient (**C**). *P < 0.05, **P < 0.01 and ****P < 0.0001.

Our paired pre-RR and RR samples, by definition, included specimens from patients with leprosy who developed a RR after the initiation of MDT. Since patients can spontaneously develop RR and present to the clinic prior to diagnosis and antibiotic treatment, we evaluated antimicrobial responses in untreated patients across the spectrum of leprosy. To do so, we sampled the following patient groups: the RR pre-MDT group, comprising individuals who experienced a RR episode prior to MDT initiation (*n* = 12); the T-lep group, consisting of 10 untreated, borderline-tuberculoid (BT) patients; the borderline-lepromatous (BL) group, consisting of 6 BL patients from the pre-RR group; and the L-lep group, consisting of 5 untreated, lepromatous-lepromatous (LL) patients along with LL1 and LL2 patients from the original pre-RR group (Supplemental Table 2). An additional differential gene expression analysis between the original RR group versus the new RR pre-MDT group showed that only 8 genes were differentially expressed (*Padj* < 0.05) (*HTRA3*, *GFPT2*, *GNA14*, *MEDAG*, *OSMR*, *ANGPTL8*, *PLA2G2A*, and *SLC39A14*) between these groups, suggesting that, regardless of when the RR was triggered, the episodes progressed similarly. Dimension reduction analysis showed a clear separation of T-lep and L-lep samples (Supplemental Figure 6), whereas some of the RR pre-MDT and BL specimens were localized between the T-lep and L-lep groups or clustered with the T-lep group. Hierarchical clustering performed with the expression values of the 64-gene antimicrobial response signature indicated coclustering of most T-lep and RR pre-MDT samples due to the higher antimicrobial gene expression when compared with the BL and L-lep groups, which clustered together (Figure 4A). The RR pre-MDT samples RR6 and RR10 that clustered with the BL and L-lep groups were notable for their low expression of the 64-gene antimicrobial response signature, while the sample BL4 clustered with the RR pre-MDT and T-lep groups.

To correlate the level of expression of the 64-gene antimicrobial response signature with clinical measures of bacillary load in patients with leprosy who had not received MDT, we computed the *z* score of the antimicrobial response signature for each patient. We noted higher antimicrobial response signature *z* scores for the T-lep (mean = 0.53, SEM ± 0.25) and RR pre-MDT (mean = 0.36, SEM ± 0.19) groups when compared with the L-lep (mean= –0.93, SEM ± 0.22) group (Figure 4B). Although not significant, the BL group (mean = –0.53, SEM ± 0.56) had a lower average antimicrobial response signature *z* score compared with scores for the T-lep and RR pre-MDT groups. We next examined the correlation between antimicrobial gene expression and various measures of bacillary load, including *RLEP* (*M*. *leprae*-specific repetitive element) gene expression (49), the skin bacillary index (SBI), and the bacillary index (BI). In the groups without treatment, *RLEP* expression was positively correlated with the patients’ SBI values (*r* = 0.87, *P* < 0.0001) (Supplemental Figure 7A) and inversely correlated with their 64-gene antimicrobial response signature *z* scores (Figure 4C) (*r* = –0.71, *P* < 0.0001). Furthermore, the 64-gene antimicrobial response signature *z* scores were inversely correlated with both the BI (*r* = –0.62, *P* < 0.0001) and SBI (*r* = –0.56, *P* = 0.0005) values (Supplemental Figure 7, B and C). We then conducted this analysis exclusively on the genes regulated by each UPR of the 64-gene antimicrobial response signature and observed that the individual antimicrobial gene programs induced by IL-17 (*n* = 32 genes), IFN-γ (*n* = 35 genes), TNF (*n* = 54 genes), and IL-1β (*n* = 44 genes) were also inversely correlated with the patients’ bacterial burden (Supplemental Figure 7, D–G). Taken together, these results indicate that expression of the 64-gene antimicrobial response signature correlated with the CMI and host defense response against *M*. *leprae*.

### Identification of molecules with direct antimicrobial activity in RR skin lesions.

**Figure 5:**
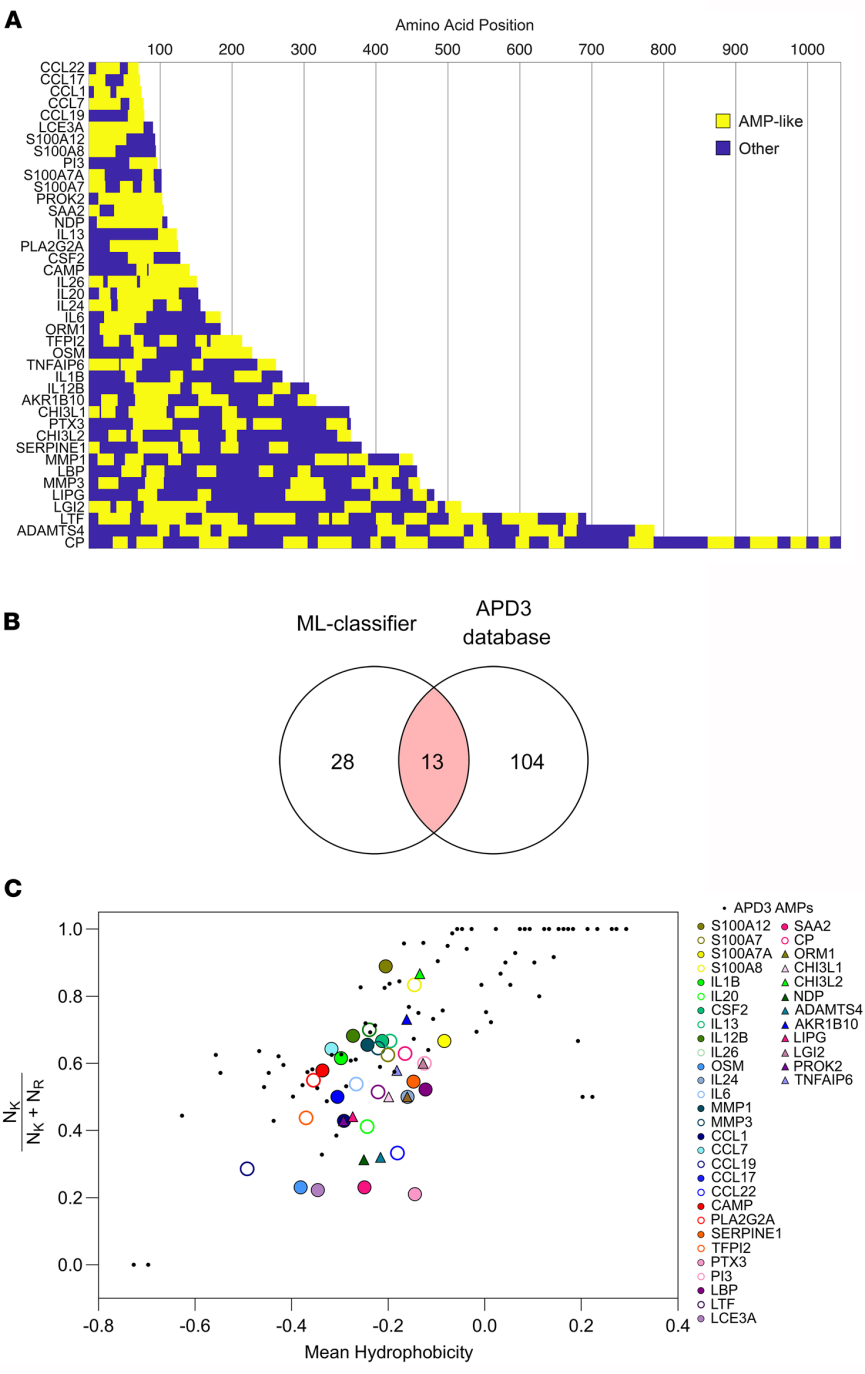
Genes upregulated in RR skin lesions encode proteins with membrane-active AMP motifs. (**A**) Graph displaying the amino acid position of the AMP-like motifs (yellow) identified along the protein sequence encoded by the RR-upregulated genes. (**B**) Venn diagram depicting the overlap between the 41 RR genes with AMP-like motifs and the human AMPs in the APD3 database (*n* = 117). (**C**) Evaluation of cationic and hydrophobic content of the AMP-like motifs detected in 41 RR antimicrobial molecules (colored circles and triangles) shown in a plot of lysine (*K*) to arginine (*R*) ratio = *N_K_*/(*N_K_* + *N_R_*) versus the mean hydrophobicity, together with known α-helical AMPs from the APD3 database (black circles).

We widened the scope of our RR transcriptome antimicrobial analysis using a machine-learning–based membrane activity prediction tool (50) to identify sequences of antimicrobial proteins with predicted membrane-permeating properties or AMP-like motifs within the 200-gene RR-upregulated signature. We evaluated the RR-upregulated genes that encoded proteins known to be “antimicrobial,” “secreted,” or located in the “extracellular matrix” according to the UNIPROT database annotation, restricting our analysis to 66 of the 200 RR genes (Supplemental Data File 4). We identified 41 RR-upregulated genes that encoded proteins with AMP-like motifs (Figure 5A). These genes have known defined roles in innate and adaptive immune responses, comprising 9 cytokines (*IL1B*, *IL6*, *IL13*, *IL20*, *IL24*, *IL26*, *OSM*, *IL12B*, and *CSF2*), 5 chemokines (*CCL1*, *CCL7*, *CCL17*, *CCL19*, and *CCL22*), 2 growth factors (*NDP* and *PROK2*), 4 S100 proteins (*S100A7*, *S100A8*, *S100A12*, and *S100A7A*), 8 acute-phase inflammatory molecules (*CP*, *LBP*, *LTF*, *PI3*, *PTX3*, *SAA2*, *CAMP*, and *ORM1*), 4 enzymes (*LIPG*, *PLA2G2A*, *AKR1B10*, and *SERPINE1*), 1 enzyme inhibitor (*TFPI2*), 6 tissue repair/remodeling proteins (*CHI3L1*, *CHI3L2*, *ADAMTS4*, *MMP1*, *MMP3*, and *TNFAIP6*), 1 neural signaling molecule (*LGI2*), and 1 epidermal structural protein (*LCE3A*). Thirteen (*CAMP*, *CCL1*, *CCL17*, *CCL19*, *CCL22*, *IL26*, *LTF*, *PI3*, *PLA2G2A*, *S100A12*, *S100A7*, *S100A7A*, and *SAA2*) of the 41 identified molecules were reported in the Antimicrobial Peptide Database 3 (APD3) (51) (Figure 5B), supporting the reliability of the machine-learning classifier in predicting and identifying membrane-permeating peptide sequences. Despite having known antimicrobial activity, *KRT6A* and *RNASE2* were not included in the machine-learning classifier analysis because of our initial selection criteria.

We cross-validated our machine-learning classifier results against a previously reported AMP amino acid composition analysis known as the “saddle-splay” curve (52). The curve states an empirical relationship between the lysine-to-arginine ratio and mean hydrophobicity of a peptide to obtain antimicrobial membrane activity based on a dataset of 299 known AMPs. Our analysis confirmed that the AMP-like motifs within each of the 41 RR sequences exhibited an amino acid composition comparable to that of the reference curve (Figure 5C). Hence, given the congruency between the 2 independent analyses, the identified AMP-like motifs may generate the topological negative Gaussian curvature used by classical AMPs to disrupt membranes rich in negative curvature lipids. The identification of 28 potentially novel antimicrobial protein candidates with membrane-permeating properties expressed in RR skin lesions, aside from the 13 already known (51), gives further insight into the rich and complex host antimicrobial response that arises during leprosy’s RR.

Altogether, our analysis of genes encoding proteins with potential antimicrobial activity expressed in RR lesions identified 64 in the Gene Cards antimicrobial database and 41 with predicted membrane-permeating activity, in total comprising 77 unique genes (Supplemental Data File 5). Of these, 15 genes were found in the direct APD3 data base (51): *CAMP*, *CCL1*, *CCL17*, *CCL19*, *CCL22*, *IL26*, *KRT6A*, *LTF*, *PI3*, *PLA2G2A*, *RNASE2*, *S100A12*, *S100A7*, *S100A7A*, and *SAA2*. Twelve genes have been shown to participate in mycobacteria infection control, including *CAMP*, *IL26*, and *CSF2* (Supplemental Data File 5). We further focused on the antimicrobial activity of 4 proteins that, to our knowledge, have not been shown to kill mycobacteria directly: CCL17, CCL19, S100A7, and S100A8. Of these, S100A8 is absent from the APD3 database (51), having been identified here as a membrane-permeating protein by the machine-learning classifier.

### Validation of S100A7, S100A8, CCL17, and CCL19 expression and antimicrobial activity against mycobacteria.

**Figure 6:**
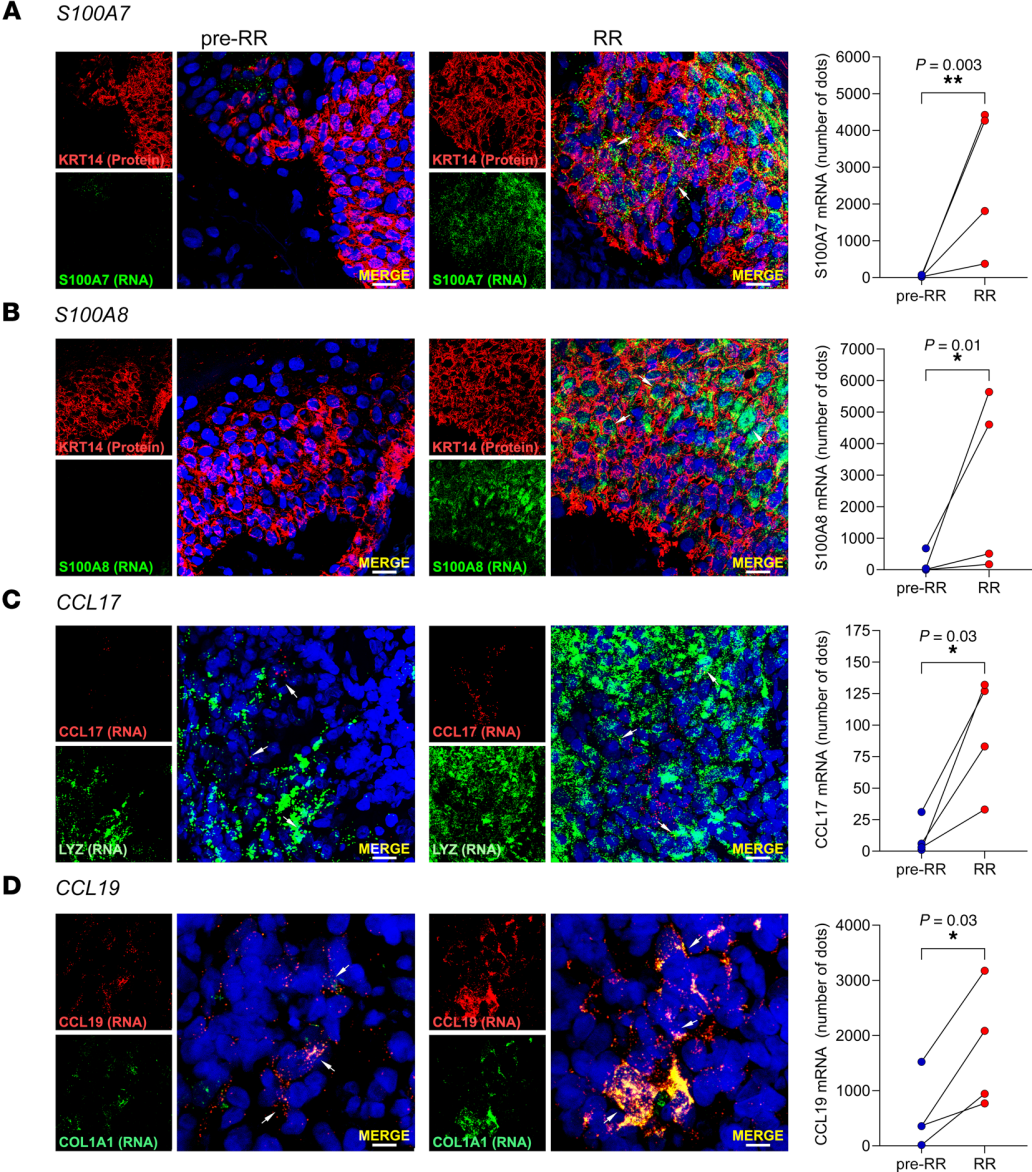
RNA-FISH shows antimicrobial gene expression in RR and pre-RR skin lesions by different cell populations. (**A**) RNA-FISH of *S100A7* (green) and staining for keratin 14 (KRT14) protein (red) in 1 representative pair of RR and pre-RR skin lesions (BL4/RR.BL4). *S100A7* RNA dot quantification (number of dots) was performed on 4 pairs of RR and pre-RR skin lesions. (**B**) RNA-FISH of *S100A8* (green) and protein staining of KRT14 (red) in 1 representative pair of RR and pre-RR skin lesions (BL5/RR.BL5). *S100A8* RNA dot quantification (number of dots) was performed on 4 pairs of RR and pre-RR skin lesions. (**C**) RNA-FISH of *CCL17* (red) and *LYZ* (green), a macrophage marker, in 1 representative pair of RR and pre-RR skin lesions (BL3/RR.BL3). *CCL17* RNA dot quantification (number of dots) was performed on 4 pairs of RR and pre-RR skin lesions. (**D**) RNA-FISH of *CCL19* (red) and *COL1A1* (green), a fibroblast marker, in 1 representative pair of RR and pre-RR skin lesions (BL4/RR.BL4). *CCL19* RNA dot quantification (number of dots) was performed on 4 pairs of RR and pre-RR skin lesions. Cell nuclei were stained with DAPI (blue). Images were acquired with a Leica TCS SP8 Digital Light Sheet microscope, and RNA dot quantification was performed using ImageJ. Scale bars: 10 μm; original magnification, ×630 (**A**–**C**) and ×630 with ×3 zoom (**D**). Statistical analyses were performed in GraphPad Prism 9.12 using the ratio paired *t* test (**A** and **B**) or paired 2-tailed *t* test (**C** and **D**). *P < 0.05 and **P < 0.01.

We first corroborated gene expression in RR versus pre-RR samples by real-time quantitative PCR (qPCR) (Supplemental Figure 8, A–D), showing a significant correlation with the RNA-Seq data (Supplemental Figure 8, E–H). Next, we validated the cell sources of these antimicrobial genes, previously determined by scRNA-Seq (18), in the RR and pre-RR specimens by RNA fluorescence in situ hybridization (RNA-FISH). We performed RNA-FISH on 4 paired skin lesions using specific mRNA probes along with probes or antibodies against specific cell population markers. Our results showed the presence of *S100A7* and *S100A8* mRNA in KRT14^+^ keratinocytes along the epidermis and in the hair follicles, and these genes were more strongly expressed in RR versus pre-RR lesions (Figure 6, A and B, and Supplemental Figure 9). We confirmed the expression of *CCL17* mRNA in myeloid cells by codetection in lysozyme-positive (*LYZ*^+^) cells activated macrophages (53), which were more numerous in the RR skin lesions (Figure 6C). The expression of *CCL19* mRNA in fibroblasts was validated in cells coexpressing type I collagen (*COL1A1*), which was more strongly detected in the dermis of RR versus pre-RR skin lesions (Figure 6D). Negative and positive controls were performed for each skin lesion evaluated by RNA-FISH (Supplemental Figures 10 and 11).

**Figure 7:**
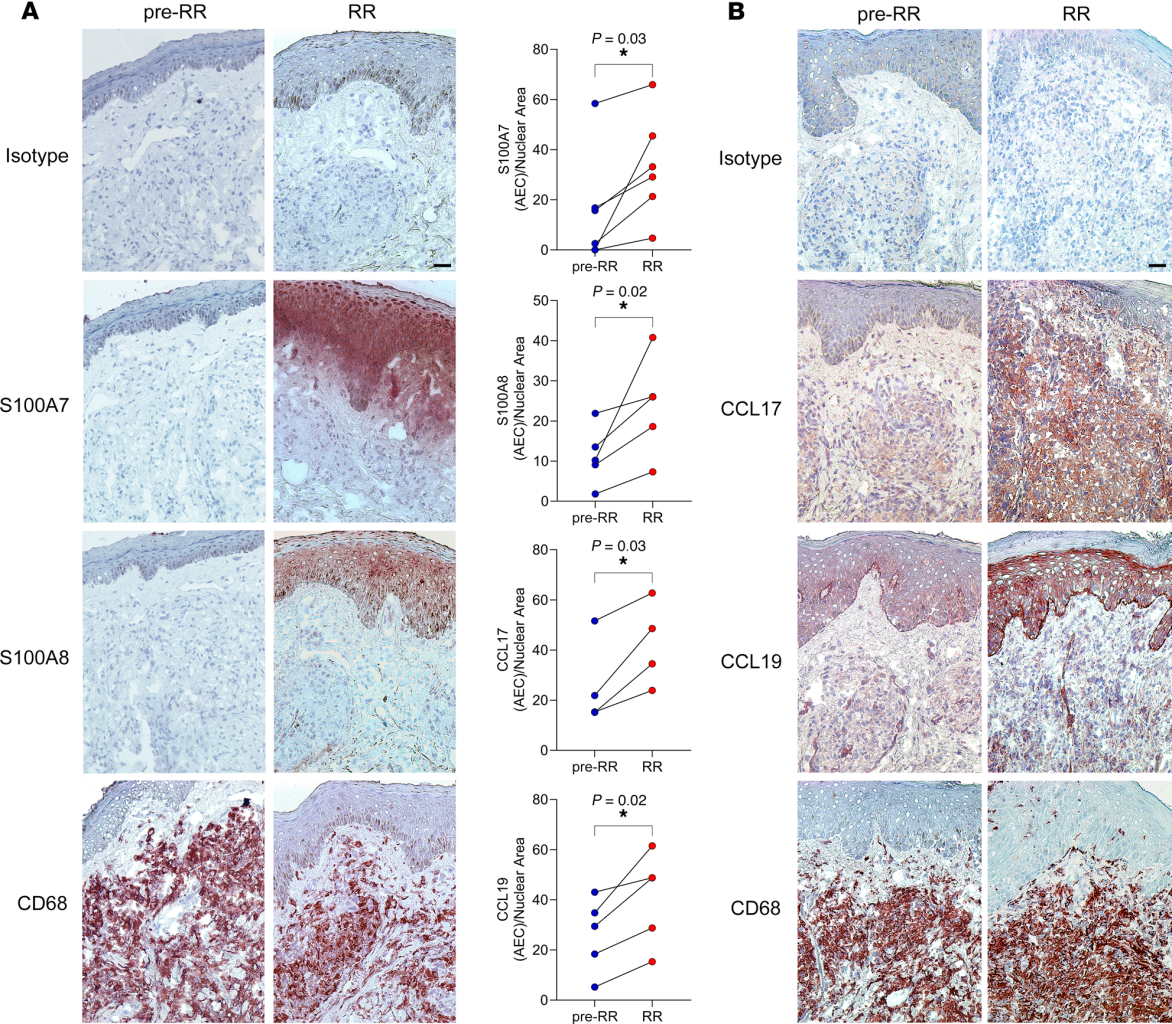
Protein expression of S100A7, S100A8, CCL17, and CCL19 in RR and pre-RR skin lesions. (**A**) S100A7 and S100A8 protein expression in a representative pre-RR and RR skin lesion pair (LL1/RR.LL1) evaluated by IHC. (**B**) CCL17 and CCL19 protein expression in a representative pre-RR and RR skin lesion pair (BL4/RR.BL4) evaluated by IHC. CD68, a macrophage marker, was used as a positive control. Graphs show quantification of S100A7 (*n* = 6 pairs), S100A8 (*n* = 5 pairs), CCL17 (*n* = 4 pairs), and CCL19 (*n* = 5 pairs) staining 3-amino-9-ethylcarbazole (AEC)/nuclear area. Image J plugin ImmunoRatio was used for quantification, and staining was visualized and images were acquired using a Leica microscope (Leica 250). Scale bars: 25 μm; original magnification, ×200. Statistical analyses were performed in GraphPad Prism 9.12 using the paired *t* test (S100A7 and CCL19) or ratio paired, 2-tailed *t* test (S100A8 and CCL17). *P < 0.05.

We assessed the protein expression of S100A7, S100A8, CCL17, and CCL19 in RR versus pre-RR skin lesions by immunohistochemistry (IHC). We observed that, in agreement with the scRNA-Seq and RNA-FISH results, S100A7 and S100A8 were more highly expressed in RR skin lesions when compared with pre-RR specimens, and their expression was concentrated on the epidermis (Figure 7A). S100A7 and S100A8 secretion by human keratinocyte cultures was also detected after stimulation with recombinant human IL-17, TNF, or IFN-γ, which are upstream regulators of the RR antimicrobial gene signature (Supplemental Figure 12). Both CCL17 and CCL19 protein expression levels were also higher in RR skin lesions when compared with levels in the pre-RR samples, with CCL17 present in the dermis in the same region as CD68^+^ macrophages and CCL19^+^ staining in cells scattered in the dermis and epidermis (Figure 7B).

**Figure 8:**
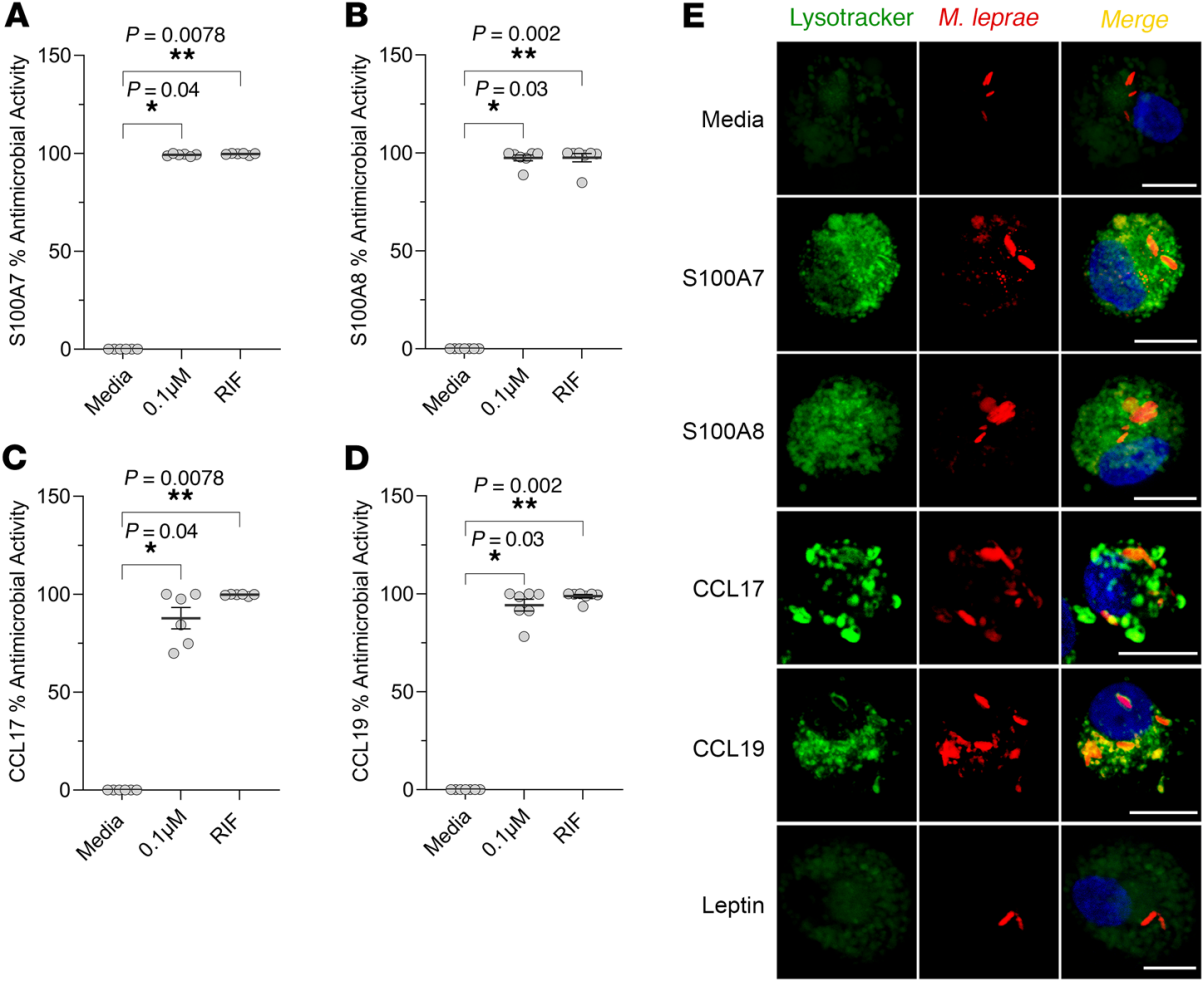
S100A7, S100A8, CCL17, and CCL19 show antimicrobial activity against *M*. *leprae* in infected human macrophages. (**A**–**D**) MDMs from healthy donors were infected overnight with *M*. *leprae* at a MOI of 5:1, followed by addition of 0.1 μM recombinant human S100A7, S100A8, CCL17, and CCL19 for 4 days. *M*. *leprae* viability was assessed by qPCR, and the percentage of antimicrobial activity was calculated by assigning 100% bacteria viability to the media control. Rifampin (10 μg/mL) (RIF) was added as a positive control. (**E**) Lysosome acidification was assessed by LysoTracker staining (green) in MDMs previously stimulated with 0.1 µM recombinant human S100A7, S100A8, CCL17, and CCL19 for 1 hour and then infected with M. leprae labeled with PKH26 (red) at a MOI of 5:1 over night. Leptin (0.1 μM) was used as a negative control. Images were captured using a Leica TCS SP8 Digital Light Sheet Microscope. DAPI (blue) was used to stain the nuclei. Scale bars: 10 μm; original magnification, ×630 with ×4 zoom. Statistical analyses were performed in GraphPad Prism 9.12 using the Friedman test followed by Dunn’s multiple-comparison test (**A**–**D**). Data represent the mean ± SEM (*n* = 6 for **A** and **C**) and (*n* =7, **B** and **D**). *P < 0.05 and **P < 0.01.

We investigated the antimicrobial activity of S100A7, S100A8, CCL17 and CCL19 proteins against *M*. *leprae* in human macrophages). We infected human monocyte-derived macrophages (MDMs) with *M*. *leprae* at a MOI of 5:1, yielding an average infection rate of 75% of the cultured macrophages (Supplemental Figure 13). We added S100A7, S100A8, CCL17, and CCL19 (0.1 μM) to the cultures and evaluated bacteria viability by qPCR after 4 days. Following titration assays, we selected rifampin as the positive control at a final concentration of 10 μg/mL (Supplemental Figure 14A). Our results showed that S100A7 (mean = 99.2%, SEM ± 0.23), S100A8 (mean = 97.4%, SEM ± 1.5), CCL17 (mean = 87.7%, SEM ± 5.4), and CCL19 (mean = 94.1%, SEM ± 3.0) exerted antimicrobial activity against *M*. *leprae* in cultured human macrophages, comparable to rifampin and notably higher than the approximately 40% reduction previously reported (27) for IL-26 at a higher concentration (2 μM) (Figure 8, A–D).

The antimicrobial activity against *M*. *leprae* was abrogated by denaturation of the proteins prior to their addition to infected cultures (Supplemental Figure 14, B–E). Additional assays using leptin (0.1 μM) as a negative control showed no antimicrobial activity against *M*. *leprae*, indicating the specificity of S100A7, S100A8, CCL17, and CCL19 activity (Supplemental Figure 14F). Additionally, staining with viability dyes confirmed that these proteins did not affect the viability of human macrophages (Supplemental Figure 15). Addition of S100A7, S100A8, CCL17, and CCL19 to MDMs infected with *Staphylococcus aureus* also led to a reduction of the bacterial load in the macrophage cultures (Supplemental Figure 16A). Furthermore, in *M*. *leprae*–infected macrophages stimulated with S100A7, S100A8, CCL17, and CCL19, PKH26-labeled bacilli colocalized with LysoTracker staining, showing that the bacteria in acidified phagolysosomes had signs of disintegration when compared with control media and the negative control with 0.1μM leptin (Figure 8E). These findings suggest that S100A7, S100A8, CCL17, and CCL19 led to a reduction in *M*. *leprae* viability in infected macrophages.

**Figure 9:**
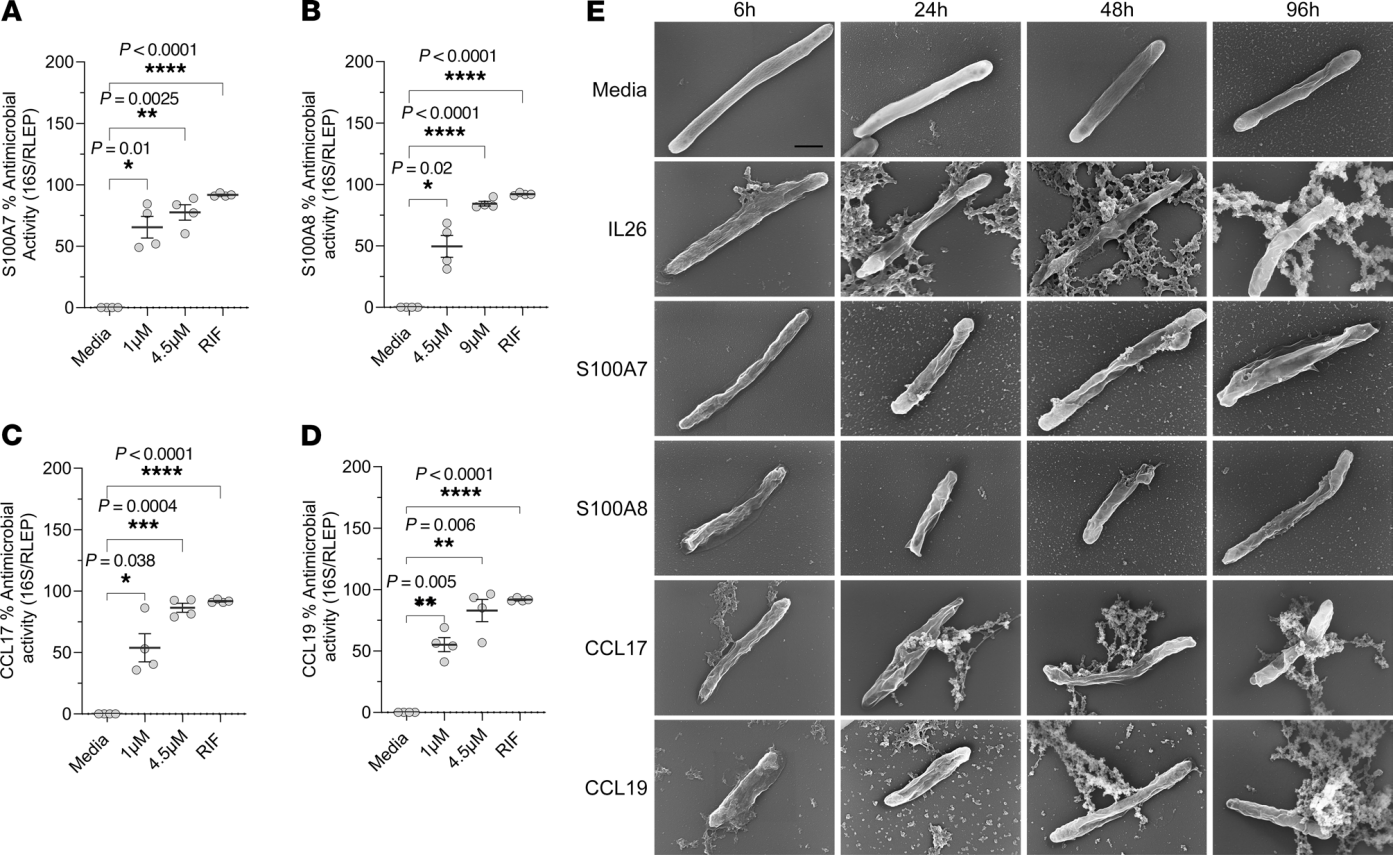
S100A7, S100A8, CCL17, and CCL19 show direct antimicrobial activity against *M*. *leprae*. (**A**–**D**) Different concentrations of recombinant human S100A7, S100A8, CCL17, and CCL19 were added to *M*. *leprae* (2 × 10^6^ bacilli) in 7H9 broth with 10 mM sodium phosphate, pH 7.2, for 72 hours. Bacteria viability was assessed by qPCR, and rifampin (10 μg/mL) (RIF) was used as a positive control. (**E**) S100A7 (4.5 μM), S100A8 (9 μM), CCL17 (4.5 μM), and CCL19 (4.5 μM) were added to *M*. *leprae* (15 × 10^6^ bacilli) in 7H9 broth with 10 mM sodium phosphate, pH 7.2, for 6, 24, 48, and 96 hours, and bacteria morphology was evaluated by scanning electron microscopy. IL-26 (10 μM) was used as a positive control. Scale bar: 500 nm; original magnification, ×100,000. Statistical analyses were performed in GraphPad Prism 9.12 using repeated-measures of 1-way ANOVA with the Geisser-Greenhouse correction and Dunnett’s multiple-comparison test (**A**–**D**). Data represent the mean ± SEM (*n* = 4). **P < 0.01, ***P < 0.001, and ****P < 0.0001.

Since these molecules interact with cell receptors to perform their classical functions, the antimicrobial activity observed in infected macrophages may have been indirectly triggered through receptor-ligand interactions. Therefore, to corroborate the machine-learning classifier analysis, we tested the potential of S100A7, S100A8, CCL17, and CCL19 to directly kill mycobacteria by performing antimicrobial assays with *M*. *leprae* and *M*. *smegmatis* in axenic cultures. For the antimicrobial assays in axenic cultures, we performed dose titration experiments and observed direct antimicrobial activity using recombinant human protein concentrations 10–200 times higher than those used in the *M*. *leprae*–infected macrophage assays. Our results indicated that S100A7, S100A8, CCL17, and CCL19 could significantly decrease the viability of *M*. *leprae* in axenic cultures, with the higher concentrations inducing antimicrobial activity comparable to that of rifampin (Figure 9, A–D). Similar experiments with autoluminescent *M*. *smegmatis* (54) and the mc(2)155 strain showed that S100A7, S100A8, CCL17, and CCL19 exerted direct antimicrobial effects on these cultures (Supplemental Figure 17, A–H). Assays conducted in axenic cultures of *S*. *aureus* showed that S100A7, S100A8, CCL17, and CCL19 could also directly kill Gram^+^ bacteria (Supplemental Figure 16, B–E).

We performed scanning electron microscopy to visualize the distinct morphological changes on the bacterial membranes of *M*. *leprae*, *M*. *smegmatis*, and *S*. *aureus* after direct exposure to S100A7, S100A8, CCL17, and CCL19. IL-26 was used as a positive control because of its direct antimicrobial activity against mycobacteria (27, 29). In the absence of antimicrobial proteins, *M*. *leprae* had a rod-shaped morphology and an intact cell surface at all time points, with a smoother membrane texture at 6 hours and 24 hours and signs of corrugation at 48 and 96 hours, likely due to the bacteria’s poor survival in axenic cultures. Conversely, membrane rupture and cytoplasmic leakage could be observed on the bacteria exposed to CCL17 and IL-26 at as early as 6 hours, and to CCL19 at as soon as 48 hours, with more pronounced damage observed at later time points. *M*. *leprae* bacilli exposed to S100A7 and S100A8 showed signs of severe surface wrinkling and roughening at as early as 6 hours, with pronounced corrugation but no obvious signs of cytoplasmic leakage at the time points evaluated (Figure 9E).

Similar membrane alterations seen in *M*. *leprae* were also observed in *M*. *smegmatis* cultures after incubation with S100A8 for 6 hours, as well as with CCL17, CCL19, and IL-26 for 24 hours. In contrast, incubation of *M*. *smegmatis* with S100A7 for 6 hours revealed signs of membrane rupture and cytoplasmic leakage, which were not present at any time point in the *M*. *leprae* assay with S100A7 (Supplemental Figure 17I). Scanning electron microscopic images of *S*. *aureus* axenic cultures after exposure to S100A7, S100A8, CCL17, and CCL19 for 3 hours revealed membrane rupture and cytoplasmic leakage associated with antimicrobial activity (Supplemental Figure 16F). Taken together, our results suggest that these antimicrobial molecules contributed to host defense during *M*. *leprae* infection, either by targeting infected macrophages or by directly interacting with the bacilli during the RR.

## Discussion

Antimicrobial effector mechanisms, which are crucial components of both innate and adaptive immunity, play a vital role in combating intracellular bacterial infections, including infection with *M*. *leprae*, the etiologic agent of leprosy. The disease has as a spectrum of clinical manifestations that correlate with the immune response, yet this spectrum is also dynamic, as patients may undergo a RR. In this study, we conducted a longitudinal analysis of dynamic changes in the host transcriptome in lesions harvested from patients before and during a RR, identifying 77 antimicrobial genes upregulated in RRs. Our findings revealed the dynamic emergence of an antimicrobial gene program during RRs as part of the host immune response, correlating with a reduction of bacterial burden in patients.

The development of RRs in patients with multibacillary leprosy marks a transition from a permissive immune environment that facilitates bacterial persistence to a state of enhanced cell-mediated immunity (13), often associated with a decline in the bacteriological index (10, 11, 55). The longitudinal design of our study enabled us to assess the dynamic emergence of host innate and adaptive immune responses required to combat the infection, effectively controlling for individual variability, as each participant served as their own control. We identified a signature of 200 genes upregulated in RR versus pre-RR skin lesions involved in innate and adaptive pathways contributing to CMI such as “response to type II interferon” and “positive regulation of IL-12 production,” 64 of which are implicated in antimicrobial responses according to the Gene Cards database, including 12 with known antimicrobial roles in mycobacterial infection. An upstream regulator analysis of the 64-gene antimicrobial response signature showed the involvement of both innate (*TNF* and *IL1B*) and adaptive (*IL17A* and *IFNG*) cytokines in the induction of these antimicrobial genes. Strikingly, *IL17A* was identified as an upstream regulator for 32 of the 64 genes comprising the antimicrobial response signature. Th17 cells comprise 90% of the T cell population detected in RR skin lesions (18). In addition to confirming the role of *IFNG* and *IL1B* as regulators of antimicrobial gene expression in RRs (18), our data identified Th17 cells as the main source of *IFNG* and also as secondary contributors to *TNF* expression in RR skin lesions.

The identification of Th17 cells as major inducers of antimicrobial genes in RR lesions through the expression of *TNF*, *IFNG*, and *IL17A* provides important new insights into the role of this T cell subset in leprosy immunopathogenesis. The IL-17–induced antimicrobial gene program correlated with the reduction in viable bacilli in leprosy lesions. Previous studies have established the presence of Th17 cells in patients with leprosy, in both RR (18) and T-lep skin lesions (47, 48, 56), as well as in PBMCs of RR patients (56–58). Higher levels of IL-17 isoforms were detected in the resistant forms of leprosy (48, 59), including RR patients (60–62). Here, we identified a program of IL-17–induced antimicrobial genes that encode proteins with direct antimicrobial activity as well as proinflammatory properties that enhance the host response, potentially contributing to host defense in leprosy. In tuberculosis, caused by *M*. *tuberculosis*, Th17 cells have been shown to contribute to protective immunity, particularly in the early stages of infection (63), by playing a role in the induction of chemokines (64), the recruitment of CD4^+^ T cells (64) to the site of infection, and the formation of granulomas (65, 66). Altogether, our data further support the concept that the RR involves coordinated interactions between the innate and adaptive immune systems, where bacterial ligands activate innate antigen-presenting cells that in turn prime the adaptive T cell response.

In addition to mining a literature-based database containing genes involved in antimicrobial responses, we also used a machine-learning algorithm to predict proteins with direct antimicrobial activity. This prediction was based on the observation that antimicrobial peptides must generate a negative Gaussian curvature (NGC) to generate membrane-permeating activity (52, 67). A total of 41 genes upregulated in RR skin lesions encode proteins with predicted membrane-permeating properties. Of these, 13 have demonstrated direct antimicrobial activity against 1 or more pathogens from a broad spectrum tested (51). Of the other 28 genes, we further investigated S100A8, which forms a heterodimer with S100A9, called calprotectin, with a broad spectrum of direct antimicrobial activity (68–73), although neither protein by itself has been shown to be directly antimicrobial. We determined that among the 77 unique antimicrobial genes, S100A8, along with three additional proteins—S100A7, CCL17, and CCL19—exhibited direct antimicrobial activity against *M*. *leprae*, *M*. *smegmatis*, and *S*. *aureus* in axenic culture. By scanning electron microscopy, CCL17 and CCL19 induced bacterial membrane lysis with extrusion of cytoplasmic contents in all bacteria tested, as observed for IL-26 (28, 29) and other chemokines (74–76). S100A8 and S100A7 caused the extrusion of cytoplasmic contents in *S*. *aureus* and only surface wrinkling and corrugation in *M*. *leprae*. Given that S100 proteins can also contribute to antimicrobial responses by metal chelation (72, 73, 77), further studies are required to investigate the mechanism(s) of their antimicrobial activity against *M*. *leprae*. Thus, the approaches used here led to the identification of 4 proteins that, to our knowledge, exhibited previously unreported direct antimicrobial activity against *M*. *leprae*.

In addition to having direct antibacterial activity, AMPs can activate macrophages to kill intracellular bacteria. We found that S100A7, S100A8, CCL17, and CCL19 significantly reduced *M*. *leprae* viability within cultured human macrophages, demonstrating antimicrobial effects on infected cells that were comparable to those of rifampin. To our knowledge, only S100A8 has been reported to trigger an antimicrobial response in macrophages infected by mycobacteria such as *M*. *tuberculosis* and *Mycobacterium bovis* (78, 79). The addition of AMPs to macrophage cultures may lead to cell activation. For instance, S100A7 and S100A8 can mediate many of their biological functions through the pattern recognition receptor for advanced glycation end-products (RAGE), as well as through TLR4 80–83), leading to activation of the NF-κB pathway, autophagy, and ROS production, mechanisms known to be involved in bacterial infection control (84–90). As has been shown for IL-26, these AMPs are positively charged and could therefore bind to DNA from dying cells (28), gain entrance to intracellular compartments, and activate the stimulator of IFN genes (STING) pathway, inducing autophagy (27).

While our findings provide valuable insights into host defense mechanisms in RR skin lesions, we acknowledge some limitations of our study. Our antimicrobial assays were conducted in axenic cultures using micromolar concentrations of recombinant human proteins, a standard experimental approach (23, 27–29, 74, 75). However, physiological levels of S100A7 (91), S100A8 (92), CCL17 (93), and CCL19 (94) are typically in the pico- to nanomolar range. This discrepancy may partly reflect the use of recombinant proteins, which often lack native posttranslational modifications and may exhibit misfolding, thereby reducing their functional activity (28, 95, 96). As mentioned previously, lower concentrations of AMPs were required for antimicrobial activity against *M*. *leprae* in infected macrophages, suggesting that cell activation potentiated the antimicrobial response. It is also important to note that our assays used the *M*. *leprae* strain Thai-53 (genotype 1A), whereas the predominant strains in Brazil, where our cohort originates, are genotypes 3I and 4N (97), which may affect host responses (98). A future direction would be to perform strain-level sequencing (99, 100) to determine if there is a correlation with the host defense response. Finally, our human subject IRB limited the sampling from patients with leprosy, such that we used macrophages derived from healthy donor monocytes (3, 4, 20, 27) rather than from patients with leprosy, and tissue collection was limited to a single skin biopsy per time point per patient.

The development of a RR indicates the plasticity of both the innate and adaptive immune responses, dynamically switching from M2 to M1 macrophage phenotypes (3) and from Th2 to Th1 cytokine profiles (2), respectively, as well as from a bacterial persistence state toward the induction of antimicrobial response programs (3, 4). Our study offers a unique perspective of the dynamic CMI response during RRs, uncovering potentially new host defense mechanisms against intracellular bacteria and expanding our understanding of antimicrobial programs that may contribute to future therapeutic approaches targeting intracellular mycobacterial infection.

## Methods

### Sex as a biological variable.

Our study examined male and female patients, and similar findings are reported for both sexes.

### Leprosy biopsy specimens.

Forty-five skin biopsy specimens were collected from patients with leprosy classified by the Ridley and Jopling criteria (1966) (1) at the Souza Araújo Outpatient Unit (Oswaldo Cruz Foundation, Rio de Janeiro, Brazil) and snap-frozen in liquid nitrogen using cryogenic tubes for later sectioning and RNA extraction. A single skin lesion was collected from each patient at each time point. Clinical diagnoses were confirmed through histopathology (H&E-stained sections) and acid-fast bacilli (AFB) staining. The pre-RR group (*n* = 9) included 6 BL, 1 borderline-borderline (BB), and 2 LL biopsies collected at diagnosis, before MDT. After sample collection, patients in the pre-RR group were prescribed a 12-month course of MDT, in accordance with WHO guidelines. The RR group (*n* = 9) consisted of biopsies from the same patients at RR diagnosis, before prednisone treatment. Eight RR samples were taken during MDT, while sample RR.BL2 was collected approximately 10 months after MDT completion (Supplemental Table 1). The average time from leprosy diagnosis to RR onset among the 9 patients was 8.5 months (SEM ± 2.05).

The T-lep group (*n* = 10) included BT biopsies collected at diagnosis, prior to MDT. The L-lep group (*n* = 7) included LL lesions collected at diagnosis, also before MDT, with 2 specimens (LL1 and LL2) from the pre-RR group. The RR pre-MDT group (*n* = 12) included samples from RR patients diagnosed simultaneously with leprosy, without prior treatment for either condition (Supplemental Table 2). Finally, the BL group was composed of 6 BL samples from the pre-RR group.

### RNA-Seq of leprosy skin specimens.

Frozen leprosy skin biopsies were sectioned (4 μm, 40 sections) and lysed in RLT Buffer (Qiagen, 79216) with 1% β-mercaptoethanol and then stored at –80°C. RNA extraction and library preparation were conducted as previously described (101). Ribosomal RNA depletion and library preparation were performed with Ribozero Gold (Illumina, MRZG126) and KAPA Stranded RNA-Seq (Kapa Biosystems, KR0934) kits. Libraries were quality checked (Qubit, Bioanalyzer), barcoded, multiplexed (8 samples/lane, 10 μM/library), and sequenced on a HiSeq 4000 (Illumina, 100 bp single-end reads).

### RNA-Seq data analysis.

Sequenced reads were demultiplexed and aligned to the human genome (hg19, UCSC) using TopHat (version 2.0.6) and Bowtie2 (version 2.0.2), as previously described (102). Raw counts were generated with HTSeq (EMBL) and normalized using DESeq2 (Bioconductor). Dimensionality reduction of leprosy transcriptome data was performed with *t*-distributed stochastic neighbor embedding (*t*-SNE) on normalized counts of the most variable genes expressed in at least 1 sample, using the R package “tsne”. Differential gene expression between RR and pre-RR samples was analyzed using the paired inverted β binomial test (R package “countdata”) (103). RR-upregulated genes were identified as those with a *Padj* of less than 0.3 and a log_2_ FC of greater than 0.5 and downregulated genes as those with a *Padj* of less than 0.3 and a log_2_ FC of less than –0.5.

### Functional gene analysis.

Enrichment analysis of gene ontology (GO) terms, WikiPathways, and Reactome gene sets was performed on the genes upregulated in the RR versus the pre-RR groups using Metascape, version 3.5 (https://metascape.org/gp/index.html#/main/step1) (45).

### RR antimicrobial response gene signature analysis.

The RR antimicrobial response signature was derived from the overlap of upregulated genes in RRs with a GeneCards list of 1,693 molecules involved in antimicrobial responses and host defense (https://www.genecards.org/Search/Keyword?queryString=”antimicrobial”; accessed February 2023). UPR analysis of this signature was performed using IPA (Qiagen). The antimicrobial response gene signature score for each patient was calculated as the mean expression of genes in the signature using log_10_-normalized counts. The *z* scores were computed by subtracting the mean score and dividing by the standard deviation. Additionally, a list of human AMPs from the APD3 database (March 2023) (https://aps.unmc.edu/) was used to identify genes encoding proteins with direct antimicrobial activity (51).

### M. leprae bacillary load indices.

The *M*. *leprae* burden of the leprosy specimens was evaluated according to the BI and the SBI, which were generated by quantification of AFB in skin slit smears obtained from earlobes and skin lesion sections by Wade Fite staining, respectively, using a logarithmic scale (104, 105). The relative bacterial burden in leprosy skin lesions was also determined by qPCR of *M*. *leprae* repetitive element (*RLEP*) DNA (49).

### Cell population analysis using leprosy scRNA-Seq.

We explored the cell population source of the RR antimicrobial response signature by mining a previous scRNA-Seq dataset by mining a previous scRNA-Seq dataset (GSE151528) of untreated RR lesions (*n* = 5) and multibacillary skin lesions (*n* = 5) (18). The major cell types including T cells, myeloid cells, keratinocytes, endothelial cells, and fibroblasts were found in both groups, and *z* scores using the average expression of genes across identified cell clusters were calculated, as previously described (18). A cut-off *z* score of greater than 2 was applied to observe the specific RR antimicrobial genes for each cell type in the RR skin lesions.

### Machine-learning–based membrane activity prediction classifier.

A machine-learning–based membrane activity prediction classifier was used to discover amino acid sequences with membrane-permeating antimicrobial activity, or AMP-like motifs, in the RR gene signature as previously described (50, 106, 107). The genes of the RR-upregulated transcriptome were searched in the UniProt protein database (https://www.uniprot.org/) by gene symbol, and only the encoded proteins with the annotation keywords “secreted,” “extracellular matrix,” or “antimicrobial” were considered in the analysis. A candidate AMP-like protein-coding gene was considered for further evaluation if its median σ-score of its motifs was greater than 0.113 [or P(+1) >0.6] (Supplemental Methods).

### Amino acid composition analysis of AMPs.

We applied the “saddle-splay selection rule” to further evaluate the amino acid sequence of the RR-upregulated molecules unveiled by the machine-learning classifier (52). We compared the amino acid composition of the RR-upregulated protein-coding genes identified by the machine-learning classifier with the compositions of a set of 299 known cationic AMP sequences obtained from the APD3 database (51). We calculated the mean hydrophobicity and the lysine (*K*) to arginine (*R*) ratio using the equation *N*_K_/(*N*_K_ + *N*_R_) for each amino acid sequence. Only the amino acid composition of the predicted AMP-like motifs was used to compute such properties and for evaluation against the reference “saddle-splay curve” (Supplemental Methods).

### RNA-FISH.

RNA-FISH was performed on pre-RR and RR skin lesions using the RNAscope Multiplex Fluorescent Detection Kit v2 (ACDBio, catalog 323100) following the manufacturer’s instructions. We used probes for *S100A7* (ACDBio-C2, catalog 817121-C2), *S100A8* (ACDBio-C1, catalog 425271), *CCL17* (ACDBio-C1 catalog 468531), *CCL19* (ACDBio-C3, catalog 474361-C3), *COL1A1* (ACDBio-C2, catalog 401891-C2) and *LYZ* (ACDBio-C3, catalog 421441-C3) mRNA molecules. The RNAscope 3-Plex Positive Control Probe (catalog 320861) and the RNAscope 3-Plex Negative Control Probe (catalog 320871) were used as controls. Signal was detected using Tyramide Signal Amplification (TSA) Cyanine 3 & 5, tetramethylrhodamine (TMR), and the Fluorescein Evaluation kit (PerkinElmer, catalog NEL760001KT).

Identification of keratinocyte populations by immunofluorescence was performed as previously described (20, 27), with a cytokeratin 14 (KRT14) monoclonal antibody (Thermo Fisher Scientific, catalog MA5-11599, clone LL002) used at 2 μg/mL. Quantification analysis was performed using ImageJ (Analyze Particles, NIH) on all pairs of RR and pre-RR skin lesions evaluated. Images were acquired with a Leica TCS SP8 Digital Light Sheet microscope.

### Immunohistochemical analysis.

IHC was performed as previously described (20, 27). Monoclonal antibodies (10 μg/mL) against human S100A7 (Thermo Fisher Scientific, catalog MA5-16199, clone 47c1068), S100A8 (R&D Systems, catalog MAB4570, clone 749916), CCL19 (Thermo Fisher Scientific, catalog MA5-23833, clone 54909), CCL17 (LSBio, catalog LS-C198166, clone 1F11), and CD68 (2 μg/mL) (Dako, catalog M087629-2, clone PG-M1) were used. Monoclonal mouse IgG1 and IgG2b isotype controls (10 μg/mL) were included in every assay. Staining was visualized using a Leica microscope (Leica 250), and protein expression was quantified using the ImageJ plugin ImmunoRatio (108).

### MDMs.

PBMCs were isolated from peripheral blood using a Ficoll-Hypaque (GE Healthcare) density gradient. MDMs were generated as previously described (20), and cells were maintained at 37°C with 5% CO_2_.

### M. leprae.

Live *M*. *leprae* (unlabeled or labeled with PKH26) were provided by Ramanuj Lahiri (National Hansen’s Disease Program, Health Resources Service Administration, Baton Rouge, Louisiana, USA). *M*. *leprae* were grown in an athymic (nu/nu) mouse foot pad as previously described (109). All experiments with live *M*. *leprae* were performed at 35°C with 5% CO_2_.

### Antimicrobial assays with M. leprae–infected MDMs.

Antimicrobial experiments with *M*. *leprae*–infected MDMs were performed as previously described (20). Briefly, MDMs (5 × 10^5^) were infected with *M*. *leprae* at a MOI of 5:1 overnight in RPMI 1640 supplemented with 10% FCS without antibiotics at 35°C and 5% CO_2_. Cells were stimulated the next day with 0.1 μM recombinant human S100A7 (R&D Systems, catalog 9085SA050), S100A8 (BioLegend, catalog 719906), CCL17 (Peprotech, catalog 300-30), and CCL19 (Peprotech, catalog 300-29B). Rifampin was added as a positive control (10 μg/mL). Denatured recombinant proteins (0.1 μM) and recombinant human leptin (Peprotech, catalog 300-27) were used as negative controls. After 4 days, TRIzol reagent (Thermo Fisher Scientific) was added to the cells. RNA and DNA extraction was performed according to the manufacturer’s instructions. The viability of *M*. *leprae* was determined by qPCR (20, 27, 49). After 2^–(ΔCt)^ analysis, the ratio of *16S* to *RLEP* was calculated, and the percentage of bacteria viability was assessed relative to the media control.

### Antimicrobial assays in axenic culture.

For direct antimicrobial experiments with *M*. *leprae*, we added different concentrations of S100A7, S100A8, CCL19, and CCL17 to 2 × 10^6^ bacilli in Middlebrook 7H9 culture media supplemented with 10 mM sodium phosphate dibasic (pH 7.2). Rifampin was used as a positive control (10 μg/mL). *M*. *leprae* assays were performed for 3 days at 35°C. TRIzol was added to the pelleted bacteria, and viability was assessed by qPCR as previously described (20, 27, 49). The ratio of *16S* to *RLEP* was calculated, and the percentage of antimicrobial activity was calculated relative to the control.

### Scanning electron microscopy.

Scanning electron microscopy was performed as previously described (110). *M*. *leprae* (15 × 10^6^), *M*. *smegmatis* (5 × 10^6^), and *S*. *aureus* (5 × 10^6^) antimicrobial assays were conducted in axenic culture with different incubation durations. *M*. *leprae* were incubated for 6, 24, 48, and 96 hours at 35°C. *M*. *smegmatis* were incubated for 6 or 24 hours, and *S*. *aureus* were incubated for 3 hours at 37°C. Recombinant human IL-26 (R&D Systems, catalog 1375-IL/CF-MTO) (10 μM) was used as a positive control. Images were captured using a Zeiss Supra 40VP Field Emission Scanning Electron Microscope at an acceleration voltage of 10 kV (Supplemental Methods).

### Statistics.

Descriptive statistics for continuous variables are expressed as the mean ± SEM. Data distribution was assessed using the Kolmogorov-Smirnov test with Dallal-Wilkinson-Lillie analysis for *P* values and/or Q-Q plots. Two groups of paired samples were compared using the ratio paired, 2-tailed *t* test or paired *t* test, whereas independent groups were analyzed with 1-way ANOVA followed by Tukey’s or Dunnett’s multiple-comparison test. For correlation analyses, Spearman’s or Pearson’s coefficients were applied, depending on the data distribution. For paired samples across multiple groups, we applied the Friedman test with Dunn’s multiple-comparison test (non-normal data) or repeated-measures ANOVA with Dunnett’s multiple-comparison test (Gaussian data). Enrichment analysis of the RR transcriptome with the Gene Cards antimicrobial list was conducted using the hypergeometric distribution. Statistical analyses were performed using GraphPad Prism 9.12, with all tests (except hypergeometric) being 2 sided and significance set at a *P* value of less than 0.05.

### Study approval.

Human peripheral blood was obtained with informed consent from healthy donors (UCLA Institutional Review Board no. 11-001274). Leprosy skin specimens were obtained from the leprosy laboratory at the Oswaldo Cruz Foundation in Rio de Janeiro, Brazil. All patients with leprosy were recruited with informed consent and the approval of the IRB of UCLA or the institutional ethics committee of Oswald Cruz Foundation.

### Data availability.

Data values reported in this manuscript are provided in the Supporting Data Values file. The sequencing data generated in this study are available in the NCBI Gene Expression Omnibus (GEO) repository (GSE280021), along with additional datasets used for other analyses (GSE151528 and GSE125943).

## Author contributions

RLM supervised and conceptualized the study. PRA performed the experiments. FM, JL, PRA, and MP conducted the bioinformatics analysis. JDA, EYL, and GCLW performed the machine learning classifier analysis. ENS provided patients’ leprosy skin lesion samples. JEG, CJD, DJM, RMBT, BJDAS, GWA, LAM, JP, and BDB provided methodological and analysis support. Funding was acquired by RLM. The manuscript was written by PRA and RLM.

## Supplementary Material

Supplemental data

ICMJE disclosure forms

Supplemental data set 1

Supplemental data set 2

Supplemental data set 3

Supplemental data set 4

Supplemental data set 5

Supporting data values

## Version 1 06/26/2025

In-Press Preview

## Version 2 09/02/2025

Electronic publication
