# Supplementary material for: Dynamics of Th1/Th17 responses and antimicrobial pathways in leprosy skin lesions
Source: J Clin Invest. 2025 Jun 26;135(17):e190736. doi: 10.1172/JCI190736 (PMC12404764; doi:10.1172/JCI190736)
Supplement: Supplemental data [file jci-135-190736-s183.pdf]

SUPPLEMENTAL ONLINE MATERIALS

Dynamics of Th1/Th17 Responses and Antimicrobial Pathways in Leprosy Skin Lesions

Priscila R. Andrade[1], Feiyang Ma[2], Jing Lu[2], Jaime de Anda[3], Ernest Y. Lee[3,4], George W. Agak[1], Craig J. Dobry[5], Bruno J. de Andrade Silva[1], Rosane M.B. Teles[1], Lilah A. Mansky[1], Jonathan Perrie[2], Dennis Montoya[2], Bryan D. Bryson[6,7], Johann E. Gudjonsson[5], Gerard C. L. Wong[3], Euzenir N. Sarno[8], Matteo Pellegrini[2], Robert L. Modlin[1,9*]

[1] Division of Dermatology, Department of Medicine, David Geffen School of Medicine, University of California, Los Angeles, Los Angeles, California, United States of America.

[2] Department of Molecular, Cell, and Developmental Biology, University of California, Los Angeles, Los Angeles, California, United States of America.

[3] Department of Bioengineering, University of California, Los Angeles, Los Angeles, California, United States of America.

[4] Department of Dermatology, University of California San Francisco, San Francisco, CA, United States of America.

[5] Department of Dermatology, University of Michigan, Ann Arbor, MI, United States of America.

[6] Broad Institute of MIT and Harvard, Cambridge, MA, United States of America.

[7] Department of Biological Engineering, MIT, Cambridge, MA, United States of America.

[8] Leprosy Laboratory, Oswaldo Cruz Foundation, Rio de Janeiro, Brazil.

[9] Department of Microbiology, Immunology and Molecular Genetics, University of California, Los Angeles, Los Angeles, California, United States of America.

*Corresponding Author: Robert L. Modlin

Address: UCLA 52-121 CHS, 10833 Le Conte Avenue, Los Angeles, California, USA

Telephone: (818) 288-2343

E-mail: rmodlin@mednet.ucla.edu

SUPPLEMENTAL ONLINE MATERIALS

Supplemental Figures and Legends (17)

Supplemental Tables (2)

Supplemental Methods

Supplemental References

Additional Supplemental Materials (Uploaded separately as Excel files)

- **Supplemental Dataset 1:** Differential gene expression analysis of RR vs. pre-RR groups.
- **Supplemental Dataset 2:** Antimicrobial Gene list from Gene Cards and APD3 database.
- **Supplemental Dataset 3:** Validation of the 64-gene antimicrobial response signature.
- **Supplemental Dataset 4:** RR upregulated genes screened by the machine learning classifier.
- **Supplemental Dataset 5:** 77 antimicrobial genes identified in RR skin lesions.

Supplemental Figures and Legends

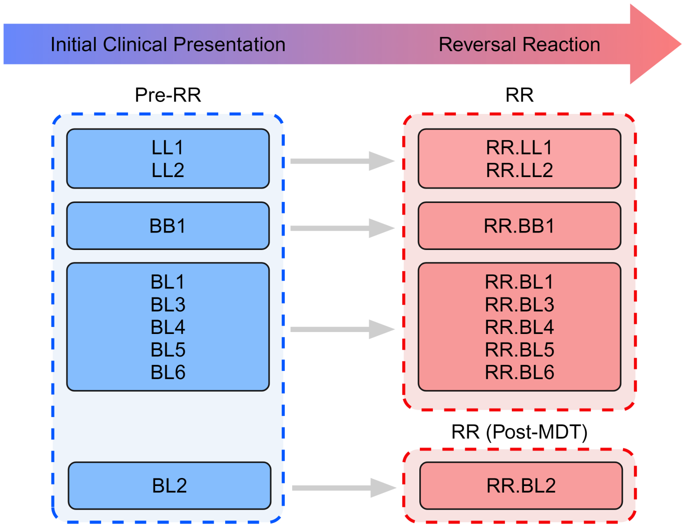

Supplemental Figure 1: Diagram of pre-RR and RR patient groups. Our study group is comprised of skin biopsy specimens obtained from nine leprosy patients at the time of leprosy initial clinical presentation (pre-RR group) and at RR clinical onset (RR group). The RR group included eight specimens from patients undergoing multidrug therapy (MDT) and one specimen collected after treatment. Created with BioRender.com.

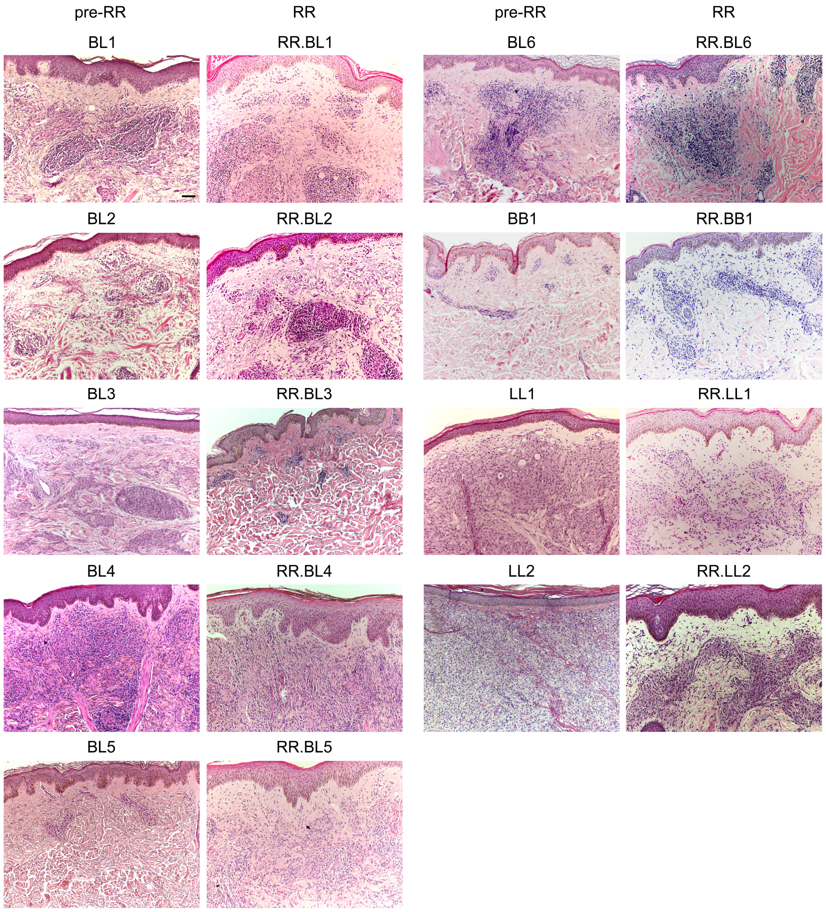

Supplemental Figure 2: Histopathology of skin biopsy specimens from pre-RR and RR groups. H&E staining was performed in leprosy skin sections of pre-RR and RR specimens and images were acquired by a Leica microscope (Leica 250). Scale bar = 50μm, original magnification 100x.

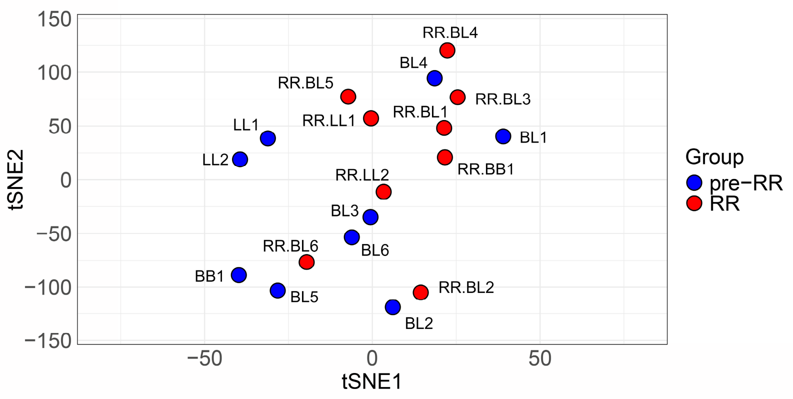

Supplemental Figure 3: t-SNE plot of pre-RR and RR transcriptome. Dimensionality reduction on the pre-RR (blue) and RR (red) transcriptomes was performed using t-SNE algorithm on normalized counts of the most variable genes expressed in at least one sample of the evaluated groups.

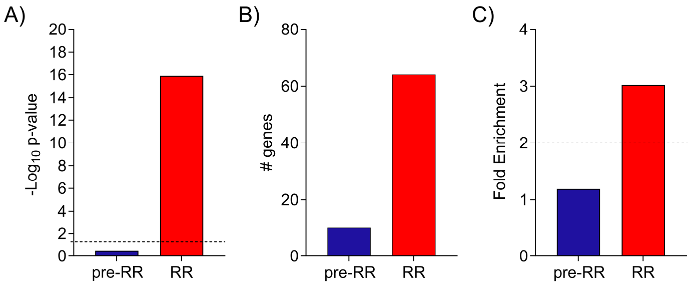

Supplemental Figure 4: Hypergeometric enrichment analysis of antimicrobial genes in the RR and pre-RR upregulated gene signature. (**A**) Enrichment -$\log_{10}$ *p*-value, (**B**) number of antimicrobial genes and (**C**) Fold enrichment of antimicrobial genes in the RR and pre-RR upregulated gene signatures calculated by hypergeometric distribution.

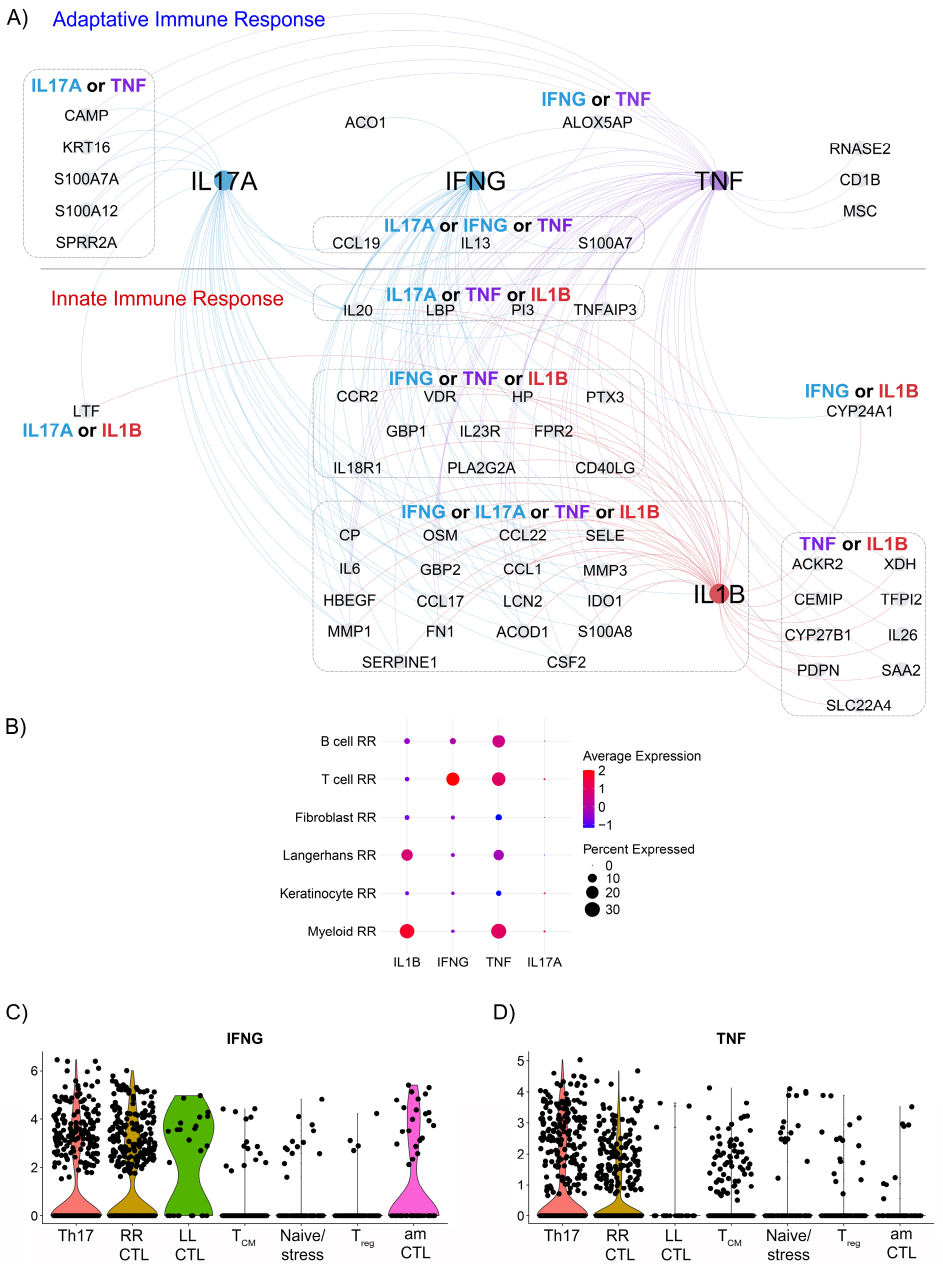

Supplemental Figure 5: Upstream Regulator analysis of the RR antimicrobial response signature. (**A**) Network depicting the RR antimicrobial genes (n=57) regulated exclusively or partially by innate immunity UPRs (red) or adaptative immunity UPRs (blue), according to the IPA UPR analysis. *TNF* is depicted as purple due to its participation in both immune responses. Network was created using Cytoscape 3.10.1. (**B**) Dot plot showing the expression of the RR UPRs by RR cell populations identified in an independent leprosy scRNA-seq data set (GSE151528). (**C** and **D**) Violin plots showing expression levels of *IFNG* and *TNF* in T cell subpopulations identified in RR skin lesions (GSE151528). T cell subpopulations included T-helper 17 (Th17), RR cytolytic T lymphocytes (RR CTL), L-lep cytolytic T

lymphocytes (LL CTL), central memory T cells (T_{CM}), naïve/stress T cells (naïve/stress), regulatory T cells (T_{reg}) and antimicrobial cytolytic T lymphocytes (amCTL).

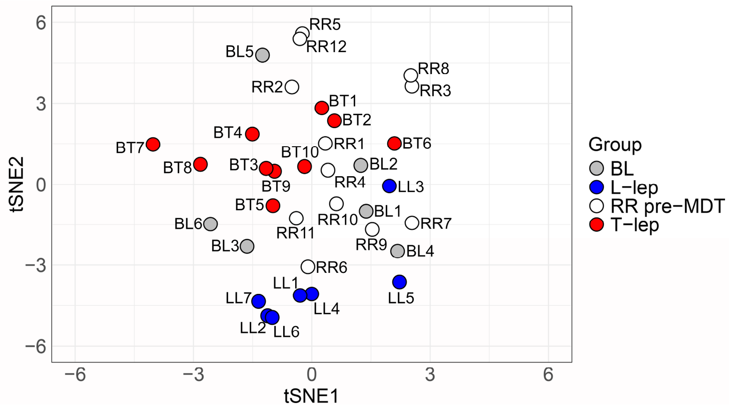

Supplemental Figure 6: t-SNE plot of T-lep, RR pre-MDT, BL and L-lep transcriptomes. Dimensionality reduction on the T-lep (red), RR pre-MDT (white), BL (grey) and L-lep (blue) transcriptomes was performed using t-SNE algorithm on the normalized counts of the most variable genes expressed in at least one sample of the evaluated groups.

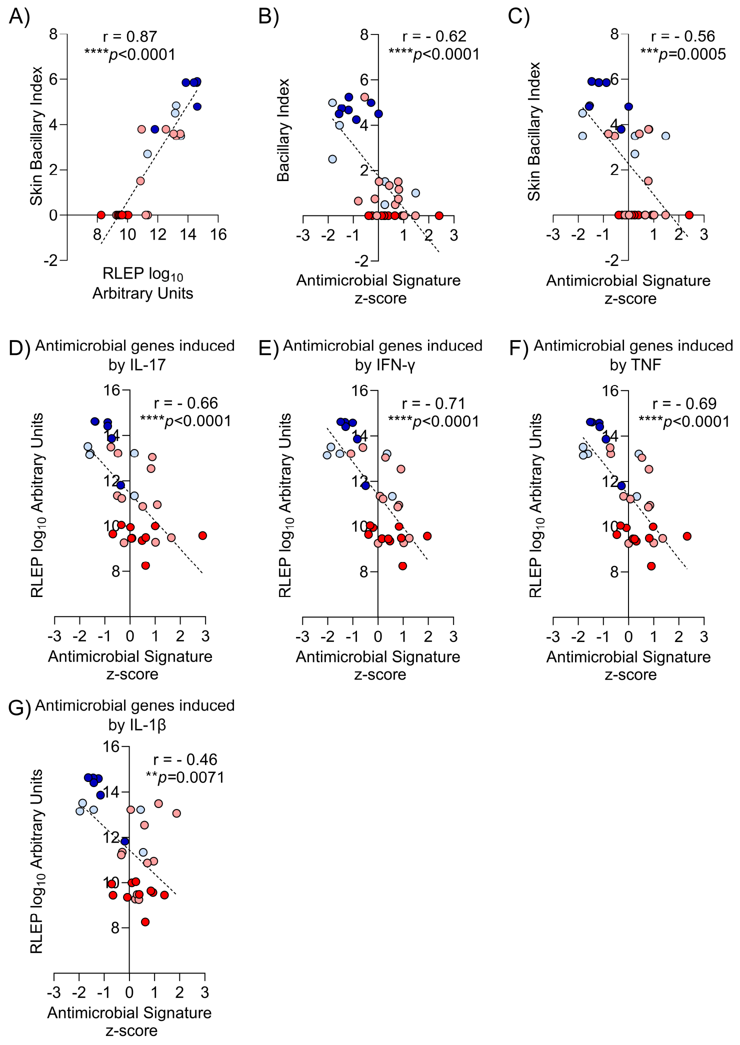

Supplemental Figure 7: Correlation analysis between antimicrobial response signature z-scores and *M. leprae* bacillary load measures. (**A**) Correlation analysis between *RLEP* expression and SBI of each patient from T-lep (red), RR pre-MDT (pink), BL (light blue) and L-lep (blue) groups. (**B** and **C**) Correlation analysis between the BI (**B**) and SBI (**C**) of each patient from T-lep (red), RR pre-MDT (pink), BL (light blue) and L-lep (blue) groups with their antimicrobial signature z-scores calculated with the normalized counts of 64 genes from the RR antimicrobial response signature. (**D-G**) Correlation analysis between *RLEP* expression and antimicrobial z-scores calculated with expression values from antimicrobial genes induced by IL-17 (n=32) (**D**), IFN-γ (n=35) (**E**), TNF (n=54) (**F**) and IL-1β (n=44) (**G**) of each patient from T-lep (red), RR pre-MDT (pink), BL (light blue) and L-lep (blue) groups. Statistical analyses were performed using Spearman correlation coefficient in GraphPad Prism 9.12 (**A-G**).

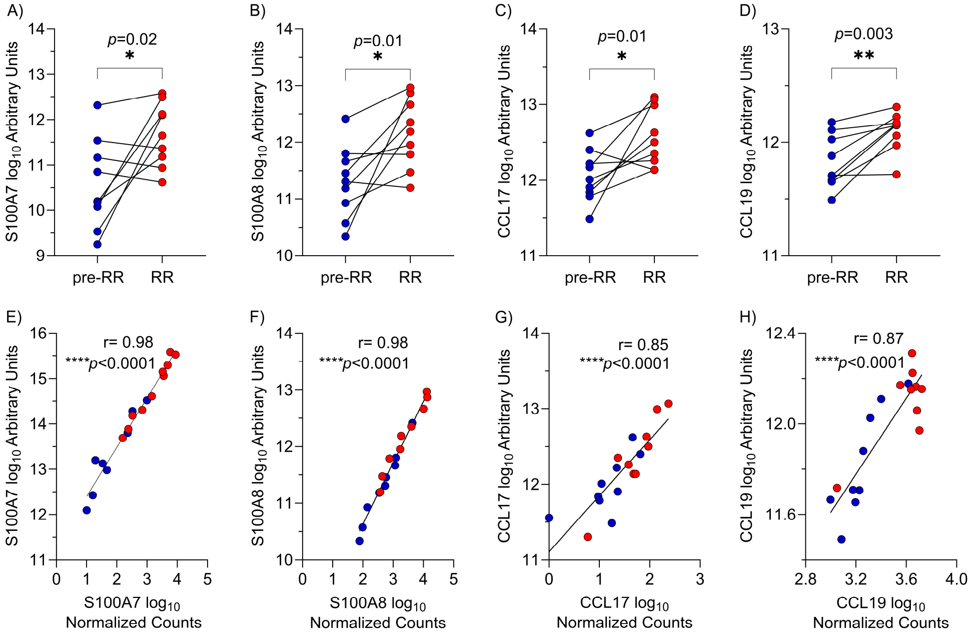

Supplemental Figure 8: Validation of RR upregulated genes by qPCR. (**A-D**) qPCR validation of *S100A7*, *S100A8*, *CCL17* and *CCL19* gene expression in RR (red) and pre-RR (blue) specimens. (**E-G**) Correlation analysis between qPCR $\log_{10}$ arbitrary units and RNA-sequencing $\log_{10}$ normalized counts (x+1) of *S100A7*, *S100A8*, *CCL17* and *CCL19* in RR and pre-RR specimens. Statistical analyses were performed in GraphPad Prism 9.12 using paired t test (**A-D**) and Pearson's correlation coefficient (**E-H**).

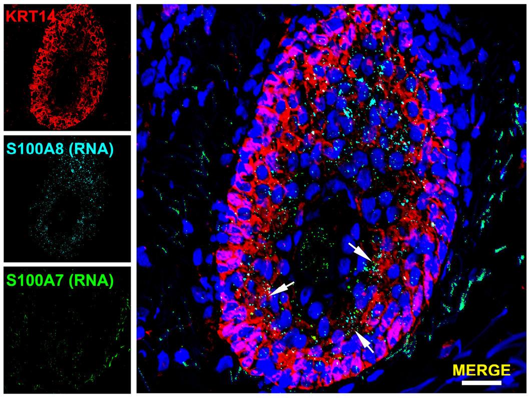

Supplemental Figure 9: **S100A7 and S100A8 RNA detection in RR skin lesion hair follicle by RNA *in situ* hybridization.** RNA-FISH of *S100A7* probe (green), *S100A8* probe (cyan) and protein staining of keratin 14 (KRT14) (red), a keratinocyte marker, in one representative RR skin lesion. Arrows indicate cells expressing S100A7 and/or S100A8. Cell nuclei were stained with DAPI (blue). Images were acquired with the Leica TCS SP8 Digital Light Sheet. Scale bars = 10µm. Magnification = 630x.

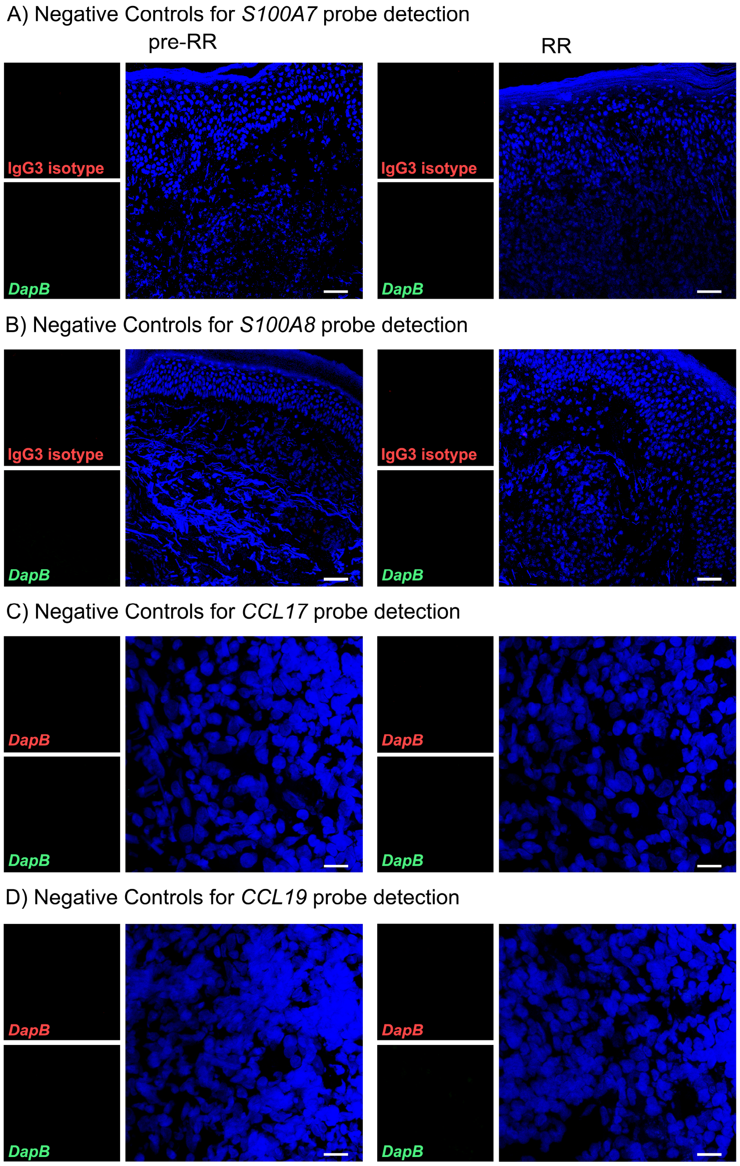

Supplemental Figure 10: **RNA *in situ* hybridization negative controls of RR and pre-RR skin lesions.** (**A** and **B**) RNA-FISH of negative controls for *S100A7* and *S100A8* probe detection and KRT14 antibody staining, displaying bacteria gene *DapB* negative control probes for channels 1 and 2 (green) and mouse IgG3 isotype control (red) in one representative pair of RR and pre-RR skin lesions. (**C**) RNA-FISH of negative controls for *CCL17* and *LYZ* probe detection, displaying bacteria gene *DapB* negative control probes for channels 1 (red) and 3 (green) in one representative pair of RR and pre-RR skin lesions. (**D**) RNA-FISH of negative controls for *COL1A1* and *CCL19* probe detection, displaying bacteria gene *DapB* negative control probes for channels 2 (green) and 3 (red) in one representative pair of RR

and pre-RR skin lesions. Cell nuclei were stained with DAPI (blue). Images were acquired with Leica TCS SP8 Digital Light Sheet Microscope. Scale bar =10μm. Magnification = 200x (**A-B**) and 630x (**C-D**).

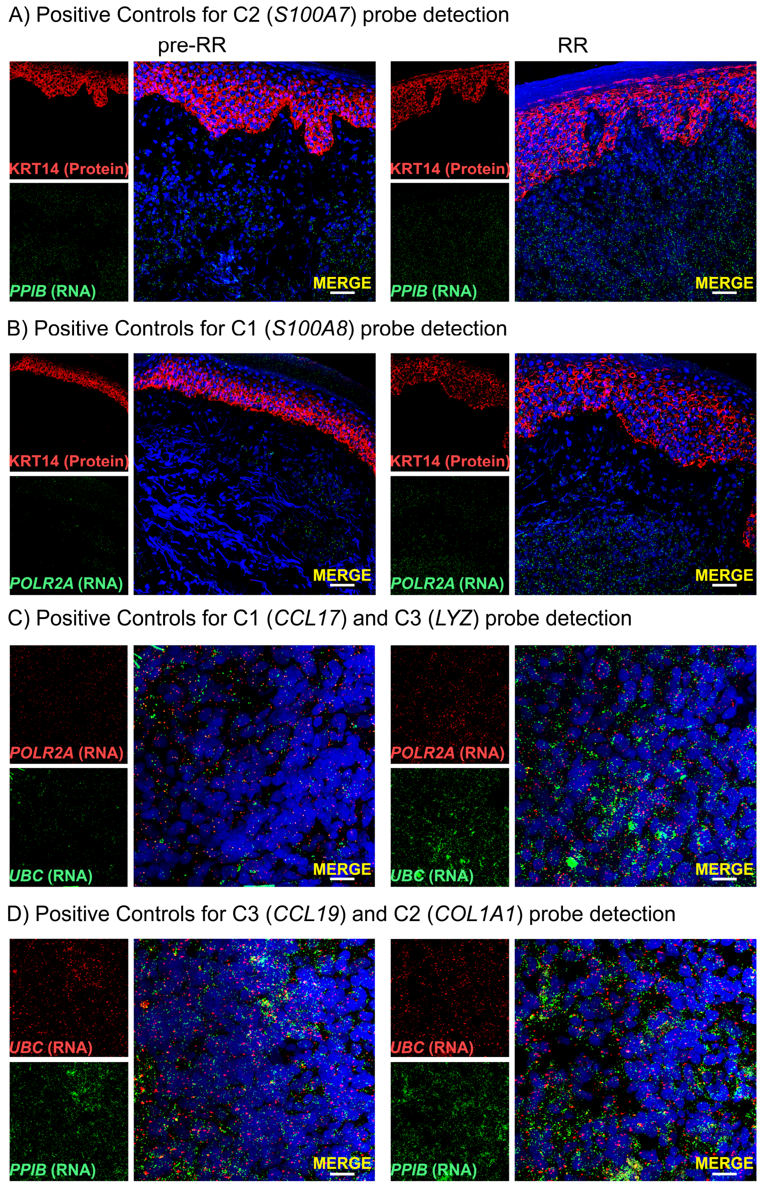

Supplemental Figure 11: **RNA *in situ* hybridization positive controls of RR and pre-RR skin lesions.** (**A** and **B**) RNA-FISH of positive control probes for channel 2 (*PPIB*) in green, corresponding to the *S100A7*-C2 probe detection, and channel 1 (*POLR2A*) in green, corresponding to the *S100A8*-C1 probe detection, with KRT14 antibody staining (red) in two representative pairs of RR and pre-RR skin lesions. (**C**) RNA-FISH of positive control probes for channel 1 (*POLR2A*) in red and channel 3 (*UBC*) in green, corresponding to the *CCL17*-C1 and *LYZ*-C3 probe detection, in one representative pair of RR

and pre-RR skin lesions. (**D**) RNA-FISH of positive control probes for channel 2 (*PPIB*) in green and channel 3 (*UBC*) in red, corresponding to the *COL1A1*-C2 and *CCL19*-C3 probe detection, in one representative pair of RR and pre-RR skin lesions. Cell nuclei were stained with DAPI (blue). Images were acquired with Leica TCS SP8 Digital Light Sheet Microscope. Scale bar = 10μm. Magnification = 200x (**A** and **B**) and 630x (**C** and **D**).

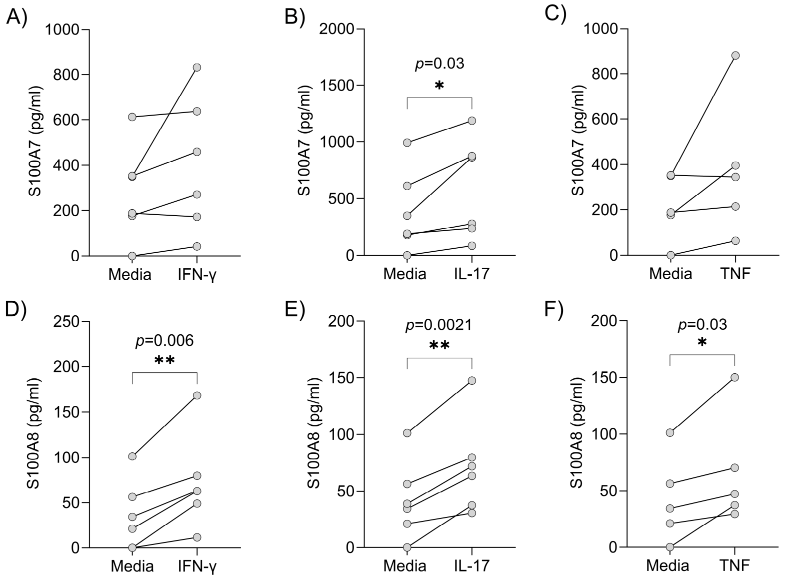

Supplemental Figure 12: Production of S100A7 and S100A8 by human keratinocyte cultures. (**A-C**) S100A7 protein secretion by human keratinocytes after stimulation with IFN-γ (5ng/ml), IL-17 (10ng/ml) and TNF (10ng/ml) after 24 hours measured by ELISA. (**D-F**) S100A8 protein secretion by human keratinocytes after stimulation with IFN-γ (5ng/ml), IL-17 (10ng/ml) and TNF (10ng/ml) after 24 hours measured by ELISA. Statistical analyses were performed in GraphPad Prism 9.12 using paired t test.

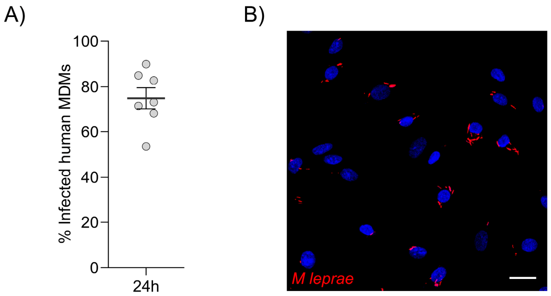

Supplemental Figure 13: *M. leprae* infection efficiency of human macrophages. (**A**) Percentage (%) of *M. leprae*-infected human monocyte-derived macrophages (MDMs) after 24 hours. (**B**) MDMs were infected with *M. leprae* labeled with PKH26 (red) at MOI 5:1 for 24 hours. Bacteria morphology was visualized using a Leica TCS SP8 Digital Light Sheet Microscope. DAPI (**blue**) was used to stain the nuclei. Scale bar = 10μm, original magnification 630x. Data represent the mean ± SEM (n=7).

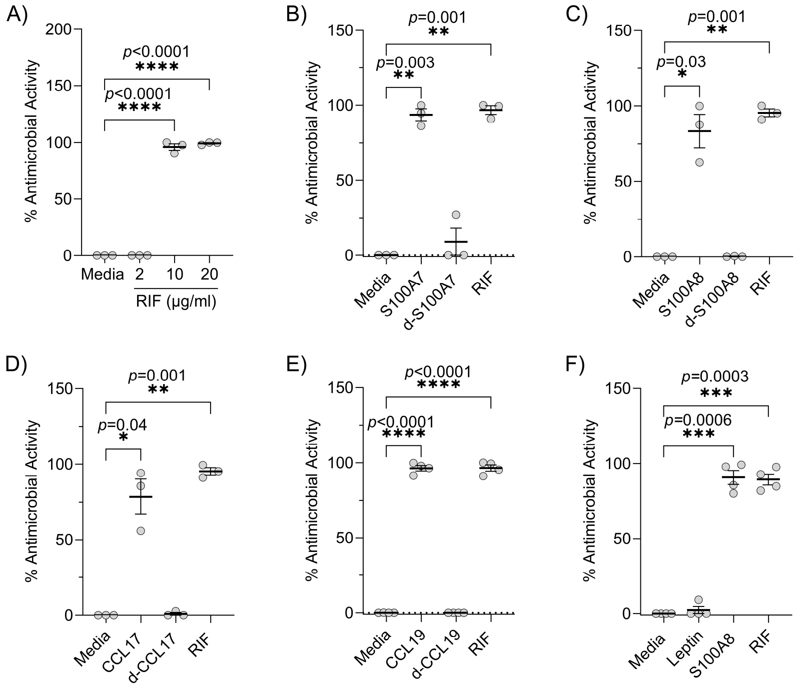

Supplemental Figure 14: Antimicrobial activity of rifampin, leptin and denatured S100A7, S100A8, CCL17 and CCL19 in *M. leprae*-infected human macrophages. Monocyte-derived macrophages (MDMs) from healthy donors were infected overnight with *M. leprae* at MOI of 5:1. (**A**) Dose titration of rifampin (RIF) was performed to determine the concentration to be used as positive control in antimicrobial assays (n=3). (**B-E**) 0.1µM of denatured (**d-**) and native recombinant human S100A7 (n=3), S100A8 (n=3), CCL17 (n=3) and CCL19 (n=4) were added to MDMs after overnight infections with *M. leprae* at MOI of 5:1 for 4 days. 10µg/ml of RIF was used as a positive control. *M. leprae* viability was assessed by qPCR and percentage (%) antimicrobial activity was calculated by assigning 100% bacteria viability to the media control. (**F**) 0.1µM of recombinant human leptin was tested for antimicrobial activity against *M. leprae* in infected MDMs (n=3), with RIF (10µg/ml) and S100A8 (0.1µM) used as positive controls. Statistical analyses were performed in GraphPad Prism 9.12 using Ordinary one-way ANOVA test with Dunnett's multiple comparisons test (**A**), repeated measures ANOVA test with the Geisser-Greenhouse correction and Dunnett's multiple comparisons test (**B-F**). Data represent the mean ± SEM.

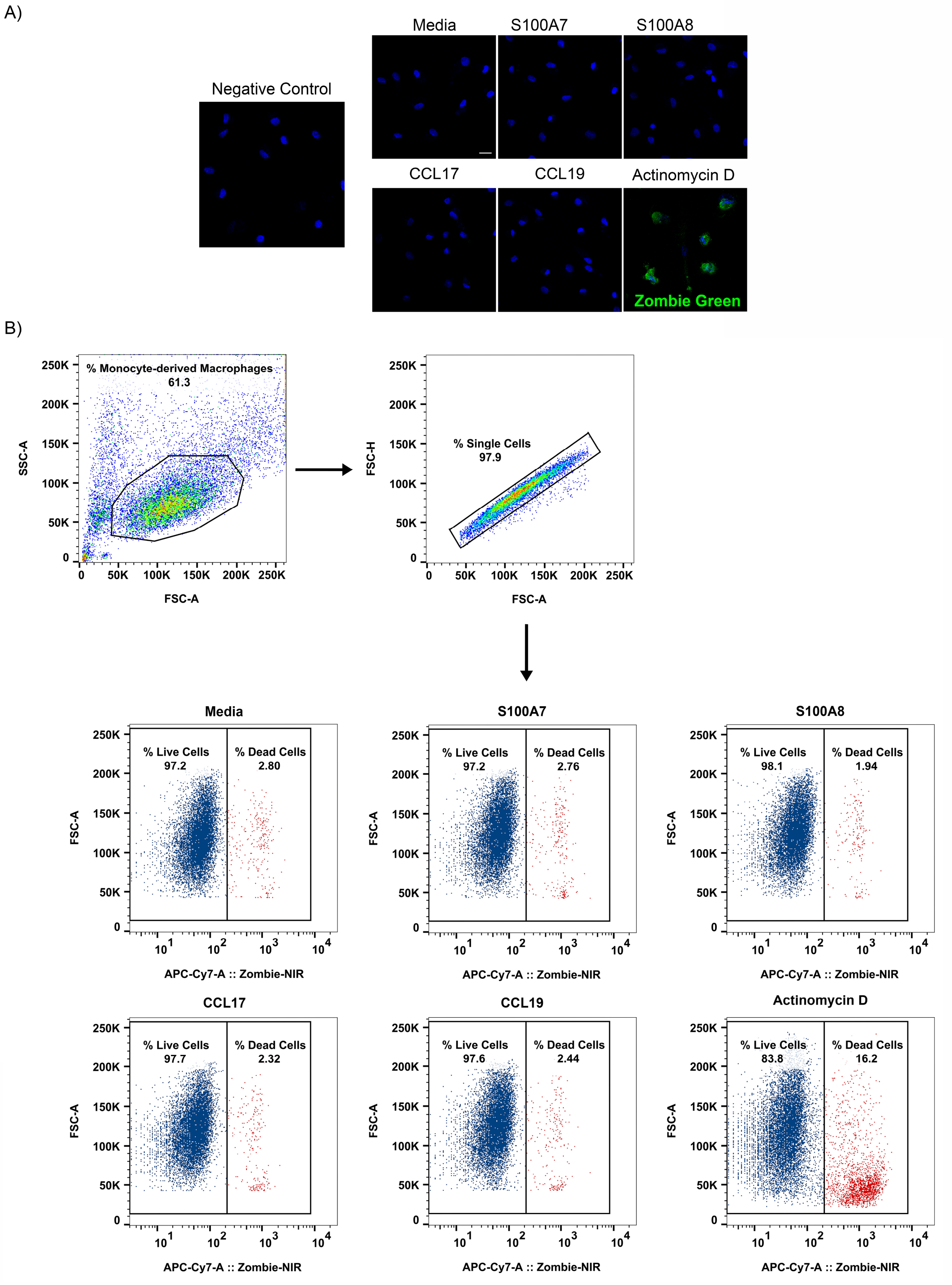

Supplemental Figure 15: Effect of S100A7, S100A8, CCL17 and CCL19 stimulation on MDM viability. Monocyte-derived macrophages (MDMs) from healthy donors (n=3) were stimulated with 0.1µM recombinant human S100A7, S100A8, CCL17, CCL19 and 500ng/ml of actinomycin D for 24 hours. (**A**)

MDMs were stained with Zombie Green™ fixable viability kit (green) and cell viability was evaluated by confocal microcopy. Images were captured using a Leica TCS SP8 Digital Light Sheet Microscope. DAPI (blue) was used to stain the nuclei. Scale bar = 10μm, original magnification 630x. (**B**) MDMs were stained with Zombie NIR™ fixable viability kit and cell viability was evaluated by flow cytometry. Samples were acquired on a Attune NxT Flow Cytometer (Thermo Scientific) and graphs were created using FlowJo software version 10.10.0.

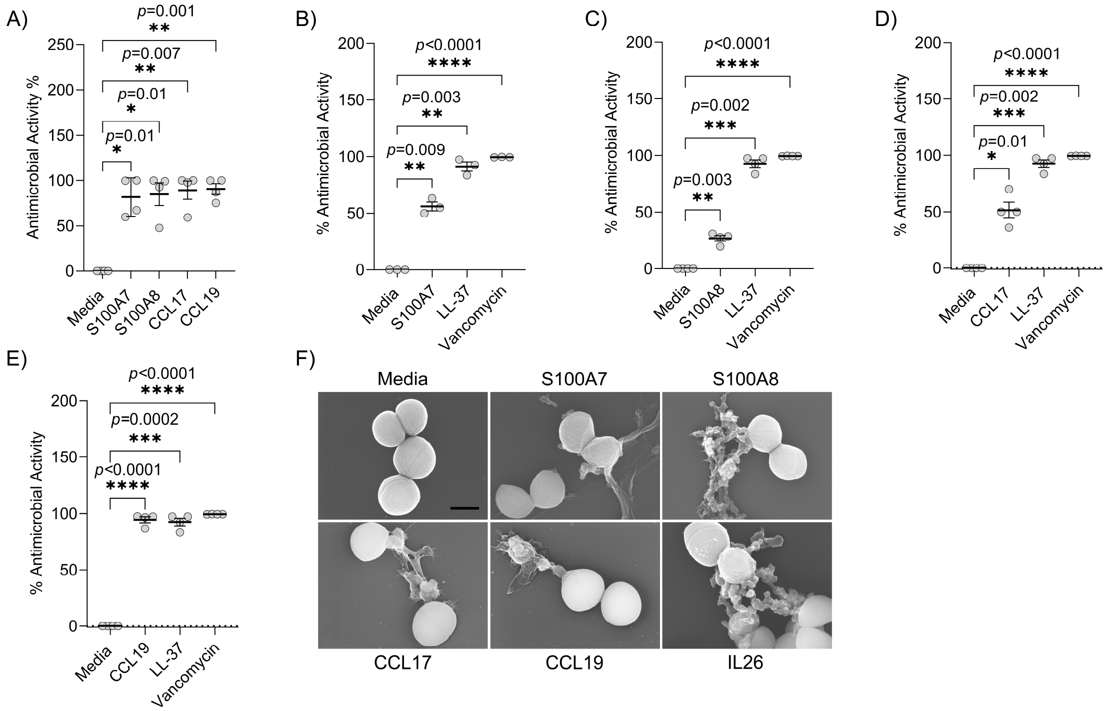

Supplemental Figure 16: Antimicrobial activity of S100A7, S100A8, CCL17 and CCL19 against *S. aureus* in axenic cultures and infected human macrophages. (**A**) Healthy donor MDMs (n=4) were stimulated with 0.1μM of S100A7, S100A8, CCL17 and CCL19 for 1 hour and infected with *S. aureus* (MOI 5:1) for 3 hours. Cells were treated with gentamycin (400μg/ml) for 1 hour, washed and incubated overnight at 37°C 5% CO2. Cells were lysed and plated onto TSA plates overnight at 37°C. Colony forming units (CFU) were enumerated the next day and percentage (%) antimicrobial activity was calculated by assigning 100% bacteria viability to the media control. (**B-E**) Recombinant human S100A7 (10μM) (n=3), S100A8 (40μM) (n=4), CCL17 (25μM) (n=4), CCL19 (20μM) (n=4), LL-37 (20μM) and vancomycin (5μg/ml) were added to *S. aureus* (4x10^5 bacilli) in 1%TSB with 10mM sodium phosphate dibasic pH=7.2 for 3 hours at 37°C. After 3 hours, each condition was plated onto TSA plates and incubated overnight at 37°C. CFU were enumerated the next day and percentage (%) antimicrobial activity was calculated by assigning 100% bacteria viability to the media control. (**F**) *S. aureus*

morphology was assessed by scanning electron microscopy. S100A7 (10μM), S100A8 (40μM), CCL17 (25μM), CCL19 (20μM) and IL-26 (10μM) were added to *S. aureus* ($5x10^6$ bacilli) in 1%TSB with 10mM sodium phosphate dibasic for 3 hours at 37°C. Images were captured using a Zeiss Supra 40VP Field Emission Scanning Electron Microscope at an acceleration voltage of 10kV. Magnification = 100,000X. Scale bar = 500nm. Statistical analyses were performed in GraphPad Prism 9.12 using repeated measures ANOVA test with the Geisser-Greenhouse correction and Dunnett's multiple comparisons test. Data represent the mean ± SEM.

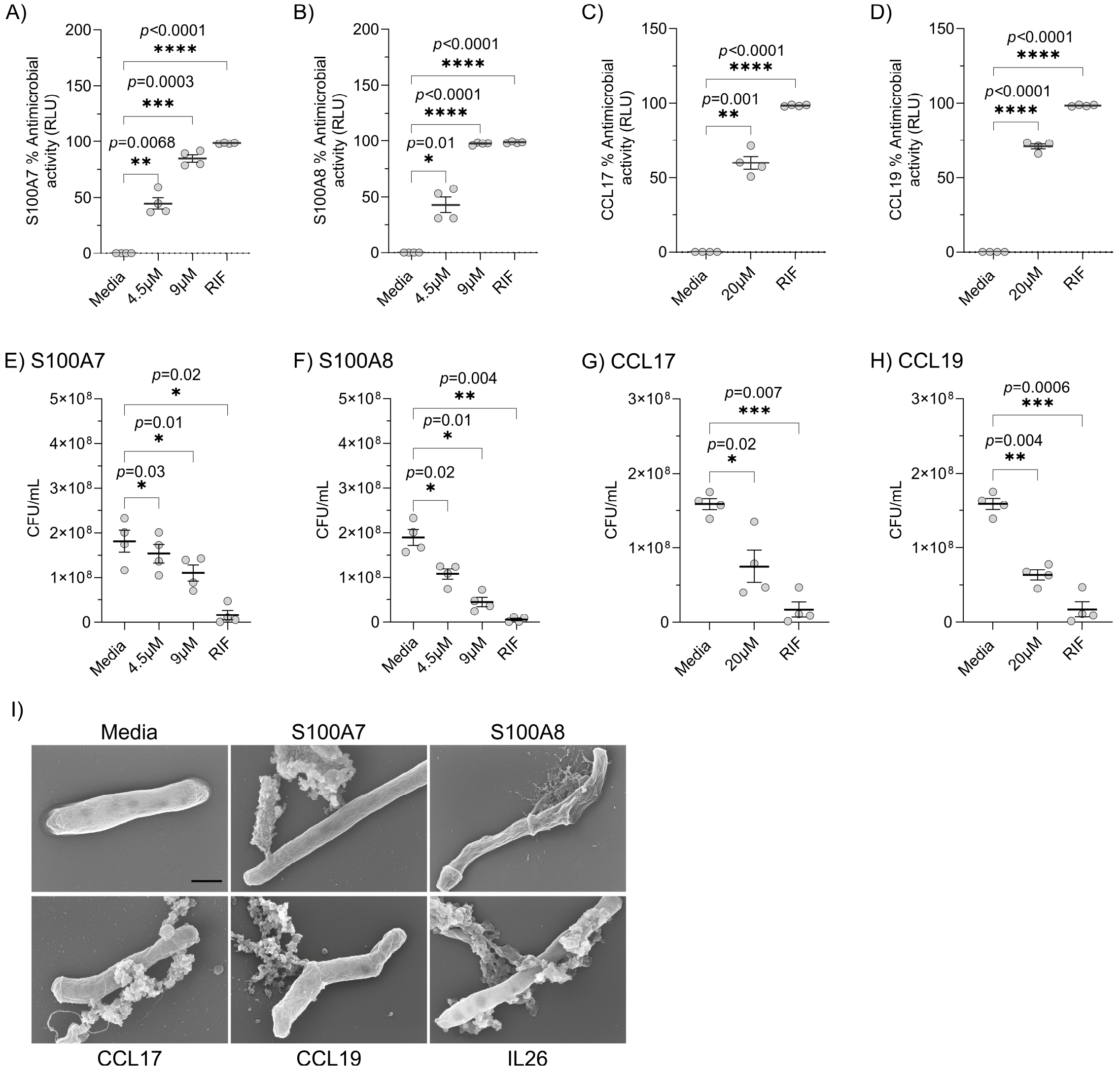

Supplemental Figure 17: Direct antimicrobial activity of S100A7, S100A8, CCL17 and CCL19 against *M. smegmatis* in axenic cultures. Different concentrations of recombinant human S100A7, S100A8, CCL17 and CCL19 were incubated with auto-luminescent *M. smegmatis* (**A-D**) or mc^2 155 strain of *M. smegmatis* (**E-H**) in 7H9 broth with 10mM sodium phosphate pH=7.2 for 24 hours. Bacteria viability

was immediately accessed by a luminometer (**A-D**) or by plating colony forming units (**E-H**). Relative light units (RLU) were used to calculate percentage (%) antimicrobial activity by comparing to media control (**A-D**) and colonies enumerated after 3 days were reported as CFU/mL (**E-H**). **I)** S100A7 (9μM), S100A8 (9μM), CCL17 (20μM) and CCL19 (20μM) were added to mc^2 155 strain of *M. smegmatis* ($5x10^6$ bacilli) in 7H9 broth with 10mM sodium phosphate pH=7.2 for 6 hours (S100A7 and S100A8) and 24 hours (CCL17 and CCL19), and bacteria morphology was evaluated by scanning electron microscopy. IL26 (10μM) incubation for 24 hours was used as a positive control for antimicrobial peptide activity. Image of the media displayed corresponds to the 24-hour time point. Magnification = 100,000X. Scale bar = 500nm. Statistical analyses were performed in GraphPad Prism 9.12 using repeated measures ANOVA test with the Geisser-Greenhouse correction and Dunnett's multiple comparisons test (**A-H**). Data represent the mean ± SEM (n = 4).

Supplemental Tables

Supplemental Table 1: Patient clinical information of pre-RR and RR groups evaluated by RNA sequencing.

						pre-RR Group							RR Group										
Patient ID	CF	Gender	Nationality	Ethnicity	Comorbidities	Sample ID	Age	BI	SBI	MDT	LT	LD date	Sample ID	Age	BI	SBI	MDT duration (months)	LT	RR date	Time from LD to RR (months)	No. RR lesions	Subsequent RR	Time between RR episodes (months)
**1**	LL	M	Brazilian	Mixed		**LL1**	31	4.7	5.9	None	0	7/12/2011	**RR.LL1**	32	4.7	3.8	5	0	11/8/2011	3.9	>20	no	NA
**2**	LL	F	Brazilian	Mixed		**LL2**	24	4.8	5.9	None	0	12/1/2011	**RR.LL2**	24	4.8	4.8	3	0	3/30/2012	4	>20	no	NA
**3**	BL	M	Brazilian	White		**BL1**	54	0.5	2.7	None	0	3/14/2011	**RR.BL1**	55	0.5	1.9	1	0	7/8/2011	3.9	<10	no	NA
**4**	BL	M	Brazilian	White	Hypertension/ Diabetes	**BL2**	60	1.5	3.5	None	0	10/20/2009	**RR.BL2**	62	0	0	Finished	0	8/22/2011	22.3	>20	yes	7.5
**5**	BL	F	Brazilian	Mixed		**BL3**	41	4	4.9	None	0	12/13/2010	**RR.BL3**	41	3.3	3.7	8	0	9/1/2011	8.7	>20	yes	8.1
**6**	BL	F	Brazilian	White		**BL4**	50	1	3.5	None	0	2/7/2011	**RR.BL4**	50	1	0	2	0	4/20/2011	2.4	>20	yes	11.2
**7**	BL	M	Brazilian	Mixed	Hypertension	**BL5**	41	2.5	4.5	None	0	2/28/2011	**RR.BL5**	42	2.5	2.8	10	0	1/25/2012	11	>20	yes	65
**8**	BL	M	Brazilian	White		**BL6**	51	5	3.5	None	0	4/25/2011	**RR.BL6**	52	2.8	4.6	10	0	2/16/2012	9.9	>20	yes	3.2
**9**	BB	F	Brazilian	Mixed		**BB1**	33	1	1	None	0	4/19/2011	**RR.BB1**	34	1	2.7	9	0	3/7/2012	10.8	>20	no	NA

CF= Clinical Form, M= Male, F= Female, LD= Leprosy Diagnosis; LT= Lepromin Test; BB= Borderline-Borderline; BL= Borderline-Lepromatous; LL= lepromatous-Lepromatous; RR= Rerversal Reaction; BI= Bacillary Index; SBI= Skin Bacillary Index; MDT= Multidrug therapy; NA= not applicable

Supplemental Table 2: Patient Clinical information of T-lep, RR pre-MDT, BL and L-lep and groups evaluated by RNA sequencing.

	Sample ID	Clinical Form	Gender	Nationality	Ethnicity	Age	BI	SBI	MDT	Lepromin Test
T-Lep Group	BT1	BT	M	Brazilian	White	53	0	0	None	6
	BT2	BT	M	Brazilian	White	44	0	0	None	4
	BT3	BT	F	Brazilian	White	69	0	0	None	0
	BT4	BT	F	Brazilian	White	60	0	0	None	4
	BT5	BT	F	Brazilian	Black	63	0	0	None	4
	BT6	BT	M	Brazilian	White	49	0	0	None	5
	BT7	BT	M	Brazilian	Black	45	0	0	None	11
	BT8	BT	F	Brazilian	White	43	0	0	None	0
	BT9	BT	F	Brazilian	Black	65	0	0	None	6
	BT10	BT	M	Brazilian	Black	66	0	0	None	5
RR pre-MDT Group	RR1	BT	M	Brazilian	White	49	0.0	0	None	0
	RR2	BB	F	Brazilian	White	58	0.5	0	None	0
	RR3	BB	M	Brazilian	White	67	0.0	0	None	0
	RR4	BT	M	Brazilian	Black	25	0.0	0	None	0
	RR5	LL	M	Brazilian	White	33	5.3	3.5	None	0
	RR6	BB	M	Brazilian	Mixed	9	0.7	3.6	None	0
	RR7	BB	M	Brazilian	Black	34	1.2	3.8	None	0
	RR8	BB	M	Brazilian	White	76	1.3	3.6	None	0
	RR9	BB	M	Brazilian	White	58	0.8	3.8	None	N/A
	RR10	BB	F	Brazilian	White	54	0.8	0	None	N/A
	RR11	RR	M	Brazilian	White	32	1.5	0	None	0
	RR12	RR	M	Brazilian	Mixed	58	1.5	1.5	None	0
BL Group	BL1*	BL	M	Brazilian	White	54	0.5	2.7	None	0
	BL2*	BL	M	Brazilian	White	60	1.5	3.5	None	0
	BL3*	BL	F	Brazilian	Mixed	41	4	4.9	None	0
	BL4*	BL	F	Brazilian	White	50	1	3.5	None	0
	BL5*	BL	M	Brazilian	Mixed	41	2.5	4.5	None	0
	BL6*	BL	M	Brazilian	White	51	5	3.5	None	0
L-Lep Group	LL1*	LL	M	Brazilian	Mixed	31	4.7	5.9	None	0
	LL2*	LL	F	Brazilian	Mixed	24	4.8	5.9	None	0
	LL3	LL	M	Brazilian	White	57	5.0	3.8	None	0
	LL4	LL	M	Brazilian	Mixed	36	5.3	5.9	None	0
	LL5	LL	F	Brazilian	White	39	4.5	4.8	None	0
	LL6	LL	M	Brazilian	White	74	4.5	4.8	None	0
	LL7	LL	F	Brazilian	Black	26	4.3	5.9	None	3

* Also part of the pre-RR group. BT= Borderline-Tuberculoid; LL= lepromatous-Lepromatous; BL= Borderline-Lepromatous; RR = Rerversal Reaction; BI = Bacillary Index; SBI = Skin Bacillary Index; MDT = Multidrug therapy; N/A = not available

Supplemental Methods

Validation of the RR antimicrobial response gene signature

We validated the expression of the RR antimicrobial response signature in the T-lep and RR pre-MDT skin lesion groups (GSE280021), as well as, in a previously published bulk RNA-seq data set of leprosy skin lesions (GSE125943) (1). Additionally, a RR signature of 66 antimicrobial genes identified by single cell RNA sequencing (scRNA-seq) (GSE151528) was also used for further validation (2). Differential gene expression analyses were conducted using DESeq2 (Bioconductor package) to derive differentially expressed gene signatures for RR vs. L-lep and T-lep vs. L-lep in the GSE280021 and GSE125943 datasets. The upregulated genes were obtained using *padj*<0.05 and $\log_2$Fold-Change>0.5. A signature of genes upregulated in RR skin lesions was generated by the union of the genes in RR pre-MDT vs. L-lep (GSE280021), RR vs. L-lep (GSE125943) and the 66 gene-RR antimicrobial signature (GSE151528). A signature of genes upregulated in T-lep skin lesions was generated by the union of the T-lep vs. L-lep (GSE280021) and T-lep vs. L-lep (GSE125943) analyses. These RR and T-lep signatures were then overlapped with the RR antimicrobial response signature found in RR vs. pre-RR analyses.

Quantitative real-time PCR (qPCR)

RR upregulated genes determined by RNA sequencing were validated by quantitative real-time PCR (qPCR). cDNA was prepared using iScript cDNA Synthesis Kit (Bio-Rad Laboratories, cat n° 1708891BUN) according to the manufacturer’s instructions. qPCR was performed using KAPA SYBR Fast qPCR kit (KAPA Biosystems, cat n° KK4602) for *S100A7* (NM_002963.4) (Forward primer: 5’- GCT GAC GAT GAT GAA GGA GAA -3’ and Reverse primer: 5’- CTT GTG GTA GTC TGT GGC TAT G -3’), *S100A8* (NM_001319196.1) (Forward primer: 5’- TGT CTC TTG TCA GCT GTC TTT C -3’ and Reverse primer: 5’- GAC ACT CGG TCT CTA GCA ATT T -3’), *CCL17* (NM_002987.3) (Forward primer: 5’- GAG TAC TTC AAG GGA GCC ATT C -3’ and Reverse primer: 5’- TCT CAA GGC TTT GCA GGT ATT -3’) and *CCL19* (NM_006274.3) (Forward primer: 5’- GGA ACT TCC ACT ACC TTC TCA TC -3’ and Reverse primer: 5’-GTC TCT GGA TGA TGC GTT CTA C -3’). Gene expression was normalized to reference

human gene *36B4* (NM_001002) (Forward primer: 5′- CCA CGC TGC TGA ACA TGC T -3′ and Reverse primer: 5′- TCG AAC ACC TGC TGG ATG AC -3′). Arbitrary units were calculated using the $2^{-(\Delta Ct)}$ method (3). Experiments were performed using the CFX96 touch real time PCR detection system (Bio-Rad Laboratories).

Primary keratinocyte cultures

Normal skin biopsy samples (4mm) were placed in Hank's balanced salt solution (Gibco, cat n°14175-095) with 0.4% dispase (Sigma-Aldrich, cat n°D4693) overnight at 4°C. Epidermis was separated from the dermis and incubated in 2ml of 1.7% Trypsin (Sigma-Aldrich, cat n°85450C) for 2 hours at 37°C. Trypsin activity was neutralized with 1ml of RPMI 1640 (Gibco, cat n°11875093) supplemented with 10% fetal calf serum (FCS) (Gibco, cat n°10438018), cells were washed and cultured with fresh Epilife™ medium with 60µM calcium (Gibco, cat n° MEPI500CA) supplemented with 2% FCS and human keratinocyte growth supplement (HKGS) (Gibco, cat n° S0015) for 3 days at 37°C with 5% CO_2. On the fourth day, media was replaced with Epilife™ medium with HKGS only and changed twice weekly for the following 2 to 4 weeks. Cells were passaged when 60% confluence was achieved and plated for experiments on the third passage.

Enzyme-linked immunosorbent assay (ELISA)

Cultures of human keratinocytes were stimulated with recombinant human TNF (10ng/ml) (R&D Systems, cat n° 210-TA-005), IFN-γ (5ng/ml) (R&D Systems, cat n° 285-IF-100) and IL-17 (10ng/ml) (R&D Systems, cat n° 314-ILB-050) for 24 hours and the collected cell free supernatants were immediately frozen at -80°C. S100A7 and S100A8 protein levels were measured in keratinocyte supernatants by the human S100A7 ELISA kit (Thermo Fisher Scientific, cat n° EH400RB) and human S100A8 ELISA kit (LS-Bio, cat n° LS-F4982-1). Absorbance (450nm) was measured using the Synergy 2 microplate reader (Biotek).

Cell viability assays

The effect of recombinant human antimicrobial proteins on the viability of MDMs was evaluated by flow cytometry and confocal microscopy. For the flow cytometry experiments, $5x10^5$ MDMs were

stimulated with 0.1µM of S100A7 (R&D Systems, cat n°9085SA050), S100A8 (Biolegend, cat n°719906), CCL17 (Peprotech, cat n°300-30), CCL19 (Peprotech, cat n°300-29B) and 500ng/ml actinomycin D (Sigma-Aldrich, cat n°A9415) for 24 hours in RPMI 1640 (Gibco, cat n°11875093) supplemented with 10% FCS (Seradigm, cat. n°FB-03) with antibiotics at 37°C with 5% CO2. Cells were detached with 1X PBS-EDTA and stained with Zombie-NIR™ fixable viability kit (Biolegend cat n° 423105) in 1X PBS (1:400) for 10 min in the dark at 4°C. Cells were washed with 1X PBS + 2% FCS and fixed in a solution of 2% PFA, 1X PBS and 1% FCS. Samples were acquired on a Attune NxT Flow Cytometer (Thermo Scientific) with the Attune Cytometric Software version 6.21. FlowJo software version 10.10.0 was used to analyze flow cytometry data and generate graphs.

For the confocal microscopy experiments $2x10^5$ MDMs were cultured in Millicell EZ slides and stimulated with 0.1µM of S100A7, S100A8, CCL17, CCL19 and 500ng/ml actinomycin D (Sigma-Aldrich, cat n°A9415) for 24 hours at 37°C with 5% CO_2. Cells were washed with 1X PBS and stained with Zombie Green™ fixable viability kit (Biolegend cat n° 423111) in 1X PBS (1:400) for 10 min in the dark at 4°C. Cells were then washed with 1X PBS + 2% FCS and fixed in a solution of 4% PFA 1X PBS for 15 minutes at 4°C. Cells were washed with 1X PBS and slides were mounted using ProLong™ Gold Antifade Mountant with DAPI. Images were captured using a TCS-SP8 Confocal Microscope with Digital Light Sheet (Leica Microsystems).

Machine learning-based membrane activity prediction classifier

The amino acid sequence for each encoded protein by the selected RR genes was screened using a previously validated machine learning classifier (4–6). Briefly, the classifier is based on a linear support vector machine (SVM) optimally trained to discriminate between antimicrobial peptides (AMPs) and decoy sequences. The machine learning classifier takes as input 12 physicochemical descriptors generated from a peptide sequence and outputs a *σ-score* specifying a likelihood that a peptide is AMP-like, or not. A positive *σ-score* indicates an AMP hit (with probability $P(+1) > 0.5$).

Genes encoding for proteins with AMP-like motifs were screened as follows: The amino acid (aa) sequences (excluding the 'signal sequence') were segmented into peptides using a moving window of

10-25 amino acids. Each sequence was individually scored by the machine learning classifier. Sequences with a σ-score>0, or P(+1)>0.5 probability, were aligned to identify consensus AMP-like motifs with the protein sequence. To obtain an amino acid position score, we first computed a mean σ-score per position for each window size, $< \sigma >_{aa}$:

$$< \sigma >_{aa} = \frac{\sum_{i=1}^{w} (\sigma_i | \sigma_i > 0)}{w}, \text{ where } w \text{ is the window size.}$$

The maximum $<\sigma>_{aa}$ score across window sizes was used to construct a maximum likelihood positional σ-score map per amino acids, $max < \sigma >_{aa}$. We discretized the scored protein map into its AMP-like motifs, i.e., consecutive positions with positive $max < \sigma >_{aa}$. A motif's σ-score was then calculated as the average $max < \sigma >_{aa}$ for all positions within its sequence. A candidate AMP-like gene encoding protein was considered for further evaluation if its median σ-score of its motifs was greater than 0.113 (or P(+1)>0.6). To impose additional rigor on the motif's boundaries, only consecutive positions with a $max < \sigma >_{aa}$ greater than 0.113 were considered an AMP-like motif, with a minimum sequence length of 15aa. If the motifs for a given protein failed to fulfill such requirements, the gene encoded protein was removed from the candidate list. Similarly, gene encoded proteins for which only certain isoforms were "secreted", or in the "extracellular matrix", were not considered if the secreted isoform did not contain the identified AMP-like motifs.

Amino acid composition analysis of antimicrobial peptides

We compared the amino acid composition of the RR upregulated protein encoding genes identified by the machine learning classifier to the compositions of a set of 299 known cationic antimicrobial peptide sequences obtained from the APD3 database (7). The mean hydrophobicity of a given peptide was calculated as:

$$\langle \text{hydrophobicity} \rangle = \frac{1}{n} \sum_{i=1}^{n} h_i$$

where n is the number of amino acids in the peptide and h_i is the hydrophobicity of the ith amino acid in the peptide using the Eisenberg consensus hydrophobicity scale (8). The lysine-to-arginine ratio $N_K/(N_K + N_R)$ is the number of lysines divided by the sum of the total number of lysines and arginines. The reference ”saddle-splay curve” was calculated as follows: The hydrophobicity range for the reference dataset was discretized into equally distributed bins. The mean $N_K/(N_K + N_R)$ ratio vs mean hydrophobicity for each set of peptides within a given bin were plotted.

To cross-validate the membrane remodeling properties of the machine learning classifier identified AMP-like motifs within the RR upregulated molecules, we calculated the mean hydrophobicity and $N_K/(N_K + N_R)$ ratio for each amino acid sequence. Only the amino acid composition of the predicted AMP-like motifs was used to compute such properties and for evaluation against the reference ”saddle-splay curve”.

Lysotracker staining of *M. leprae*-infected MDMs

To determine if *M. leprae* was being directed to acidified phagolysosomes, $2x10^5$ MDMs cultured in Millicell EZ slides with antibiotic-free media were stimulated with 0.1μM of S100A7, S100A8, CCL17 and CCL19 for 1 hour prior to infection with PKH26-labeled *M. leprae* at MOI 5 overnight at 35°C with 5% CO_2. 500nM of Lysotracker (Invitrogen™, cat n° L12492) was added to the cells for 30 minutes at 35°C with 5% CO_2. Cells were then washed with 1X PBS and fixed with 4% PFA for 30 minutes at 4°C. Cells were washed with 1X PBS and slides were mounted using ProLong™ Gold Antifade Mountant with DAPI. Images were captured using a TCS-SP8 Confocal Microscope with Digital Light Sheet (Leica Microsystems).

Staphylococcus aureus

Frozen stocks of *Staphylococcus aureus* strain DU5938 (Hla−Hlb−Hlg−) (9) were cultured in tryptic soy agar (TSA) plates (Hardy Diagnostics™, cat n° 23-001-759) overnight at 37°C. Single colonies were cultured overnight at 37°C in tryptic soy broth (TSB) (BD Bacto™ ,cat n° BD 211825) with constant shaking (225 rpm). The liquid culture was then diluted 1:100 in fresh TSB and grown to mid-log phase at 37°C with constant shaking (225 rpm).

Antimicrobial assays with *S. aureus*-infected MDMs

Antimicrobial assays with *S. aureus*-infected MDMs were performed as previously described (10). MDMs (1x10^6 cells) were pretreated with 0.1μM of S100A7, S100A8, CCL17 and CCL19 for 1 hour. MDMs were washed and infected with *S. aureus* at MOI 5:1 for 3 hours in RPMI with 10% FCS antibiotic-free media at 37°C 5% CO_2. After gentamycin treatment (400μg/ml), cells were cultured overnight at 37°C 5% CO_2. Cells were pelleted and lysed with 100μl of 0.2% saponin in 1X PBS on ice for 20 minutes. Next, 900μl of sterile 1X PBS was added to the cell lysate. Ten-fold dilutions were plated onto TSA plates and incubated overnight at 37°C. Colony forming units were enumerated and the percentage of antimicrobial activity was calculated relative to the media control.

Mycobacterium smegmatis

Frozen stocks of auto-luminescent *M. smegmatis* expressing the bacterial lux operon (11) and the mc(2)155 strain were cultured in Middlebrook 7H10 agar plates (Thermo Scientific™, cat n°R01600) for 3 days at 37°C. Single colonies were then cultured in Middlebrook 7H9 broth (BD Difco™ ,cat n° 271310) at 37°C with constant shaking (225 rpm) to mid-log phase.

Antimicrobial assays in axenic culture (*M. smegmatis* and *S. aureus*)

For direct antimicrobial experiments with mycobacteria, we added different concentrations of S100A7, S100A8, CCL19 and CCL17 to auto-luminescent or mc(2)155 *M. smegmatis* (5x10^4 bacilli) in Middlebrook 7H9 culture media supplemented with 10mM sodium phosphate dibasic (pH 7.2). Rifampin was used as a positive control (10μg/ml).

M. smegmatis assays were conducted for 24 hours at 37°C. Auto-luminescent bacteria viability was measured by a luminometer (BioTek, Synergy2™) and relative light units (RLU) were used to calculate percentage of antimicrobial activity relative to the media control. Antimicrobial activity in *M. smegmatis* mc(2)155 cultures were assessed by colony forming units (CFU) assay. After 24 hours, serial ten-fold dilutions were plated onto Middlebrook 7H10 agar plates and incubated at 37°C for 3 days.

Antimicrobial assays with *S. aureus* were performed for 3 hours at 37°C in axenic culture using 1%TSB with 10mM sodium phosphate dibasic, as previously described (12). Approximately 4x10^5

bacteria were incubated with different concentrations of S100A7, S100A8, CCL19 and CCL17. Vancomycin (5µg/ml) and LL-37 (Anaspec, cat n° AS-61302) (20µM) were used as positive controls. After 3 hours, ten-fold dilutions were plated onto TSA plates and incubated overnight at 37°C. CFU were enumerated, and the percentage of antimicrobial activity was calculated relative to the media control.

Scanning Electron Microscopy

Silicon wafers (Ted Pella Inc, cat n°16007) were washed with 100% ethanol and coated with 0.1mg/ml of poly-L-lysine hydrobromide (Sigma-Aldrich, cat n°P2636) for 2 hours at room temperature or overnight at 4°C. *M. leprae* ($15x10^6$), *M. smegmatis* ($5x10^6$) and *S. aureus* ($5x10^6$) antimicrobial assays were performed in axenic culture as previously described with different incubation times. After the assay incubation, each condition was added to a treated silicon wafer and bacteria were left to adhere for 2 hours at room temperature. Wafers were washed and fixed with a warm solution of 2.5% glutaraldehyde (EMS, cat n°16019) and 0.1M sodium cacodylate buffer pH 7.4 (EMS, cat n°11652) for 10 minutes at room temperature and then placed on ice for 1 hour. Wafers were washed five times with ice-cold 0.1M sodium cacodylate buffer pH 7.4 and post-fixed with a solution of 1% osmium tetroxide (EMS, cat n°19152) and 0.1M sodium cacodylate buffer for 30 minutes on ice protected from light. After five washes with ice-cold distilled water, wafers were dehydrated in a series of ice-cold anhydrous ethyl alcohol (EMS, cat n°15055) solutions (50%, 70%, 85% and 95%) for 3 minutes each. Lastly, samples were immersed in ice-cold 100% anhydrous ethyl alcohol three times for 3 minutes each followed by critical point drying using a Tousimis Autosamdri-810 Critical Point Dryer. Wafers were then mounted on scanning electron microscopy pin stubs (Ted Pella Inc, cat n°16111) using double sided carbon tape (EMS, cat n°77816) and sputter-coated with iridium using the Ion Beam Sputtering / Etching System (South Bay Technology). Images were captured using a Zeiss Supra 40VP Field Emission Scanning Electron Microscope at an acceleration voltage of 10kV.

Literature Search

We employed a custom Python-coded (API) to perform batch advanced literature searches in the PubMed database to identify articles describing antimicrobial effects of specific genes or encoded

proteins in mycobacterial infections. The search utilized the term "tuberculosis AND (antimicrobial OR kill)", combined with a list of individual genes and proteins formatted as "Gene X OR Protein X". For each query, the API retrieved metadata, including PubMed ID (PMID), titles, authors, abstracts, journals, and DOI links. Results were parsed into structured data frames, and the processed data was exported to an Excel file with hyperlinks to DOI references for ease of access.

Supplemental References

1. Montoya DJ, et al. Dual RNA-Seq of Human Leprosy Lesions Identifies Bacterial Determinants Linked to Host Immune Response. *Cell Rep*. 2019;26(13):3574-3585.e3.

2. Ma F, et al. The cellular architecture of the antimicrobial response network in human leprosy granulomas. *Nat Immunol*. 2021;22(7):839–850.

3. Livak KJ, Schmittgen TD. Analysis of relative gene expression data using real-time quantitative PCR and the 2(-Delta Delta C(T)) Method. *Methods*. 2001;25(4):402–408.

4. Lee EY, et al. Mapping membrane activity in undiscovered peptide sequence space using machine learning. *Proceedings of the National Academy of Sciences*. 2016;113(48):13588–13593.

5. Lee EY, et al. What can machine learning do for antimicrobial peptides, and what can antimicrobial peptides do for machine learning? *Interface Focus*. 2017;7(6):20160153.

6. Lee EY, Wong GCL, Ferguson AL. Machine learning-enabled discovery and design of membrane-active peptides. *Bioorg Med Chem*. 2018;26(10):2708–2718.

7. Wang G, Li X, Wang Z. APD3: the antimicrobial peptide database as a tool for research and education. *Nucleic Acids Res*. 2016;44(D1):D1087-1093.

8. Eisenberg D, et al. Hydrophobic moments and protein structure. *Faraday Symp Chem Soc*. 1982;17(0):109–120.

9. Nilsson IM, et al. Alpha-toxin and gamma-toxin jointly promote Staphylococcus aureus virulence in murine septic arthritis. *Infect Immun*. 1999;67(3):1045–1049.

10. Dang AT, et al. IL-26 contributes to host defense against intracellular bacteria. *J Clin Invest*. 2019;129(5):1926–1939.

11. Andreu N, et al. Optimisation of Bioluminescent Reporters for Use with Mycobacteria. *PLOS ONE*. 2010;5(5):e10777.

12. Yang D, et al. Many chemokines including CCL20/MIP-3α display antimicrobial activity. *Journal of Leukocyte Biology*. 2003;74(3):448–455.
